# Stabilisation of the RirA [4Fe–4S] cluster results in loss of iron-sensing function†

**DOI:** 10.1039/d3sc03020b

**Published:** 2023-08-22

**Authors:** Elizabeth Gray, Melissa Y. Y. Stewart, Libby Hanwell, Jason C. Crack, Rebecca Devine, Clare E. M. Stevenson, Anne Volbeda, Andrew W. B. Johnston, Juan C. Fontecilla-Camps, Matthew I. Hutchings, Jonathan D. Todd, Nick E. Le Brun

**Affiliations:** a Centre for Molecular and Structural Biochemistry, School of Chemistry, University of East Anglia Norwich Research Park Norwich NR4 7TJ UK n.le-brun@uea.ac.uk +44 (0)1603 592003 +44 (0)1603 592699; b School of Biological Sciences, University of East Anglia Norwich Research Park Norwich NR4 7TJ UK; c Department of Molecular Microbiology, John Innes Centre Norwich Research Park Norwich NR4 7UH UK; d Metalloproteins Unit, Institut de Biologie Structurale, CEA, CNRS, Université Grenoble-Alpes 71, Avenue des Martyrs, CS 10090 38044 Grenoble Cedex 9 France

## Abstract

RirA is a global iron regulator in diverse *Alphaproteobacteria* that belongs to the Rrf2 superfamily of transcriptional regulators, which can contain an iron–sulfur (Fe–S) cluster. Under iron-replete conditions, RirA contains a [4Fe–4S] cluster, enabling high-affinity binding to RirA-regulated operator sequences, thereby causing the repression of cellular iron uptake. Under iron deficiency, one of the cluster irons dissociates, generating an unstable [3Fe–4S] form that subsequently degrades to a [2Fe–2S] form and then to apo RirA, resulting in loss of high-affinity DNA-binding. The cluster is coordinated by three conserved cysteine residues and an unknown fourth ligand. Considering the lability of one of the irons and the resulting cluster fragility, we hypothesized that the fourth ligand may not be an amino acid residue. To investigate this, we considered that the introduction of an amino acid residue that could coordinate the cluster might stabilize it. A structural model of RirA, based on the Rrf2 family nitrosative stress response regulator NsrR, highlighted residue 8, an Asn in the RirA sequence, as being appropriately positioned to coordinate the cluster. Substitution of Asn8 with Asp, the equivalent, cluster-coordinating residue of NsrR, or with Cys, resulted in proteins that contained a [4Fe–4S] cluster, with N8D RirA exhibiting spectroscopic properties very similar to NsrR. The variant proteins retained the ability to bind RirA-regulated DNA, and could still act as repressors of RirA-regulated genes *in vivo*. However, they were significantly more stable than wild-type RirA when exposed to O_2_ and/or low iron. Importantly, they exhibited reduced capacity to respond to cellular iron levels, even abolished in the case of the N8D version, and thus were no longer iron sensing. This work demonstrates the importance of cluster fragility for the iron-sensing function of RirA, and more broadly, how a single residue substitution can alter cluster coordination and functional properties in the Rrf2 superfamily of regulators.

## Abstract 2

The regulator RirA senses Fe through its [4Fe–4S] cluster, which may have only three Cys ligands from the protein. Addition of a likely fourth Asp ligand stabilised the cluster, rendering it unable to sense Fe and locked into its DNA-binding state.

## Graphical abstract

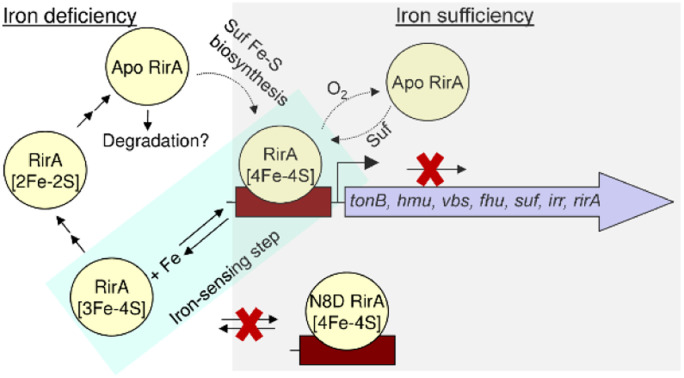

## Introduction

RirA is a broadly-distributed repressor protein that binds to *cis*-acting regulatory sequences known as iron-responsive operator (IRO) boxes^1^ near the promoters of many genes whose products are involved in iron acquisition and usage. Initially found in symbiotic bacteria of the genus *Rhizobium*,^2^ RirA orthologues occur in other closely related genera, including the important pathogens *Brucella*, *Bartonella* and *Agrobacterium* (see ref. 3). RirA belongs to the Rrf2 superfamily of transcriptional regulators, which includes NsrR (a nitric oxide-responsive regulator), IscR (a regulator of FeS biogenesis) and RsrR (a redox-sensing regulator).^4–6^ Like these proteins, RirA is a homo-dimeric iron–sulfur (Fe–S) cluster protein.^7,8^ RirA senses iron *via* its [4Fe–4S] cluster, and thus employs a mechanism that is distinct from those of the previously characterised global iron (Fe^2+^-sensing) regulators Fur and DtxR/IdeR,^9,10^ though we note the recent proposal that Fur senses iron levels through a C-terminal [2Fe–2S] cluster.^11,12^

Previous studies using UV-visible absorption and circular dichroism (CD) spectroscopies showed that, under iron limitation, [4Fe–4S] RirA undergoes conversion to a [2Fe–2S] cluster, which eventually degrades to cluster-free (apo) RirA.^8^ Electron paramagnetic resonance (EPR) spectroscopy revealed that the conversion involves the formation of a transiently stable [3Fe–4S]^1+^ cluster.^8^ However, these spectroscopic approaches did not identify any other specific cluster conversion species, which are likely EPR silent and not distinguishable by UV-visible absorbance spectroscopy.

However, using time-resolved native (non-denaturing) electrospray ionisation mass spectrometry (ESI-MS) we did identify the cluster conversion intermediates, and also elucidated the series of the molecular events involved in the conversion of [4Fe–4S] RirA cluster in response to low iron, under both aerobic and anaerobic conditions.^7^ This revealed an iron-sensing mechanism for [4Fe–4S] RirA based on an equilibrium between [4Fe–4S] and [3Fe–4S] forms, *i.e.* [4Fe–4S]^2+^ ↔ [3Fe–4S]^0^ + Fe^2+^. Under iron-replete conditions, the [4Fe–4S]^2+^ cluster form is favoured, whilst under iron depletion, the [3Fe–4S]^0^ cluster is favoured. This latter form is unstable and undergoes degradation, *via* a [2Fe–2S] form, ultimately resulting in apo RirA. The [3Fe–4S]^0^ intermediate was found, through EPR studies, to be susceptible to oxidation in the presence of O_2_, resulting in a [3Fe–4S]^1+^ form that exhibited reduced stability and an enhanced rate of degradation. The susceptibility of the [3Fe–4S]^0^ intermediate to oxidation provides the molecular basis for O_2_-sensing by RirA.^7^

*R. leguminosarum* RirA contains three Cys residues, which are conserved amongst many Rrf2 family members, including NsrR and IscR. Substitutions of these Cys residues abolished the regulatory activity of RirA in *R. leguminosarum*^2^ and *A. tumefaciens*.^13^ However, the identity of the fourth ligand to the RirA cluster is unknown. In *Escherichia coli* [2Fe–2S] IscR and *Streptomyces coelicolor* [4Fe–4S] NsrR, the clusters are coordinated by the three conserved Cys residues, plus a fourth residue: a His (His107) in the case of IscR,^14^ and an Asp (Asp8) in the case of NsrR.^15^ Neither of these ‘fourth ligand’ residues are conserved in RirA (Fig. S1†). It has been proposed that RirA may not have a fourth amino acid residue ligand to the cluster, which would account for the particular lability of one of its iron ions.^7^

Here we report further investigations of the RirA [4Fe–4S] cluster coordination. Three-dimensional modelling of RirA based on the crystal structure of [4Fe–4S] NsrR, which is the Rrf2 superfamily member most closely related to RirA,^3,16^ suggested that a fourth ligand to the cluster could be accommodated at amino acid position 8 from the other protomer, where the cluster-coordinating Asp residue is located in NsrR.^15^ In RirA, this is an Asn residue, which is not a recognised ligand of Fe–S clusters.^17^ Consequently, we sought to investigate the effect on cluster properties of substituting Asn8 in the RirA sequence. Replacement of Asn8 by Asp (as in NsrR) or Cys, to potentially generate an all-Cys coordination, did not prevent the incorporation of a [4Fe–4S] cluster. Furthermore, *in vitro* studies of these Asn8 variants revealed that they retained capacity to bind IRO box DNA, with similar affinity to that of wild-type RirA in the case of the N8D variant. Importantly, however, the [4Fe–4S] cluster of the variant RirA proteins exhibited dramatically increased stability to O_2_ and to low iron. These observations led us to hypothesise that these variants might still function as repressors of RirA-regulated genes, but with reduced capacity to respond to iron levels. *In vivo* studies of these variants in *Rhizobium* confirmed these predictions. The data support a model of RirA in which the cluster lacks a fourth amino acid residue ligand, and that this is key to the mechanism of iron sensing. This work highlights the importance of the cluster coordination for determining functional properties of Rrf2 family regulators, which exhibit significant variation in the cluster type and cluster properties within a common protein framework.^6,15,18^

## Materials and methods

### Structural modelling

**Fig. 1:**
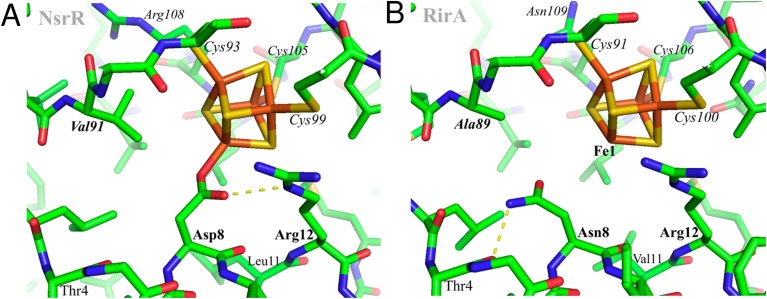
Structural modelling of [4Fe–4S] RirA. Based on (A), the structure of [4Fe–4S] NsrR,^15^ a model of the RirA cluster environment was obtained (B). Compared to Asp8 of NsrR, Asn8 of RirA is predicted to be in a different rotamer to prevent clashes between its amide group and either Arg12 or the cluster. A likely Asn8 rotamer is stabilized in RirA by a hydrogen bond to the carbonyl oxygen of Thr4, shown with the dashed line. The same side chain conformation for Asp8 would not be possible in NsrR because of its collision with Val91 (substituted by Ala89 in RirA). Substitution of Asn8 by Asp is clearly feasible, and we note the relatively few residue differences in the immediate cluster environments of NsrR and RirA, consistent with the observed spectroscopic similarities between N8D RirA and wild-type NsrR. In contrast, substitution of RirA Asn8 with Cys would not result in its coordination to Fe1 unless there was a significant protein conformational change. The expected non-protein ligand to the site-differentiated iron ion is not included in (B).

Using AlphaFold^19^ (https://github.com/deepmind/alphafold), structures were predicted for both wild-type *R. leguminosarum* RirA and for its N8D variant. However, this otherwise powerful tool does not yet allow modelling of protein-bound metal sites, probably explaining the low reliability score for the predicted protein environments of the [4Fe–4S] cluster. Therefore, the cluster environment in RirA was modelled with COOT,^20^ starting from the known structure of *Streptomyces coelicolor* NsrR^15^ and selecting connected amino acid regions positioned within 12 Å from the cluster iron and sulfur atoms (residues 3–16, 33–36, 87–91, 96–114 and 136–144 in RirA numbering, see also Fig. 1). Amino acid substitutions were applied where the amino acid sequence differed from that of *Sc*NsrR (Fig. S1†), choosing the side chain rotamer conformation giving the fewest collisions with the rest of the protein. Where necessary, a simple geometry optimization in COOT was used to remove remaining bad contacts.

### Purification of wild type and RirA variants

RirA (CAC35510) from *R. leguminosarum* strain 8401 was over-expressed in *E. coli* as previously described.^8^*rirA* genes encoding the N8D and N8C variants of RirA were synthesised by Genscript and cloned using *Nde*l and *Bam*H1 into the pET11a vector (Novagen) for protein overexpression and purification work. Both the wild-type and variants of RirA were purified as described previously.^8^ Briefly, cells were harvested by centrifugation (10 000×*g* for 15 min, 4 °C) and cell pellets were resuspended in 70 mL of buffer A (25 mM Hepes, 2.5 mM CaCl_2_, 50 mM NaCl, pH 7.5), treated with 30 μg mL^−1^ lysozyme and 30 μg mL^−1^ phenylmethane sulfonyl fluoride, and homogenized by syringe inside an anaerobic cabinet (O_2_ < 10 ppm). Suspensions were taken out, sonicated twice while on ice and under nitrogen gas flow, and returned to the anaerobic glovebox. The cell suspension was transferred to O-ring sealed centrifuge tubes (Nalgene) and centrifuged (40 000×*g*, 45 min, 1 °C) outside of the cabinet. Using an ÄKTA Prime system in the anaerobic cabinet, the supernatant was passed through a HiTrap Heparin (1 × 5 mL; Cytiva) column, washed with Buffer A until *A*_280 nm_ < 0.1, and then bound proteins eluted using a linear gradient from 0 to 100% buffer B (25 mM Hepes, 2.5 mM CaCl_2_, 50 mM NaCl, 750 mM KCl, pH 7.5). Fractions containing RirA were pooled and stored anaerobically in a freezer until needed. A small proportion (<5%) of the variant protein was truncated, as previously reported for wild-type RirA.^8^ Cluster loading for as-isolated proteins was variable, <10% for wild-type RirA,^8^ and 30–50% for the Asn8 variants.

*In vitro* cluster reconstitution to generate [4Fe–4S] wild type and variant RirA was carried out in the presence of NifS, as described previously.^21^ Gel filtration was carried out under anaerobic conditions using a Sephacryl S-100 HR 16/50 column (Cytiva), previously equilibrated with 25 mM Hepes, 333 mM KCl, pH 7.5 with a flow rate of 1 mL min^−1^. Protein concentrations were determined using the method of Bradford (Bio-Rad), with bovine serum albumin as the standard. Cluster concentrations were determined by iron assay^22^ or by using an absorbance extinction coefficient at 382 nm (ref. 8) for the wild-type RirA [4Fe–4S] cluster of 13 460 ± 250 M^−1^ cm^−1^. Apo RirA was prepared from as isolated holo protein by aerobic incubation with 5 mM EDTA overnight.

### Spectroscopic experiments

UV-visible absorbance and CD measurements were made with a Jasco V500 spectrometer and Jasco J810 spectropolarimeter, respectively. Samples were prepared in an anaerobic glovebox (O_2_ < 10 ppm) and measured in a 1 cm pathlength anaerobic quartz cuvette. To simulate low iron conditions, the soluble high affinity iron chelator EDTA (Fe^2+^-EDTA, log *K* = 14.3, Fe^3+^-EDTA, log *K* = 25.1)^23^ was used. For iron chelator and O_2_ experiments, protein samples were placed in an anaerobic cuvette, a solution of the iron chelator EDTA was added to a final concentration of 1 mM, and the cluster response was followed *via* spectroscopy. For the O_2_ experiments, protein samples were rapidly diluted with aerobic buffer (25 mM Hepes, 2.5 mM CaCl_2_, 50 mM NaCl, 750 mM KCl, pH 7.5) to give the desired O_2_ concentration, and the cluster response was immediately followed by spectroscopy.

### Mass spectrometry measurements

Reconstituted N8D and N8C RirA variants were buffer-exchanged into 250 mM ammonium acetate, pH 7.3 under anaerobic conditions using PD Minitrap G-25 (Cytiva) desalting columns. For low iron condition experiments, EDTA was added to a final concentration of 250 μM. For low iron experiments in the presence of O_2_, anaerobic protein samples in 250 mM ammonium acetate, pH 7.3, were rapidly diluted with an air-saturated buffer containing EDTA to give the desired final O_2_ and EDTA concentrations. Protein samples were infused directly (0.3 mL h^−1^), *via* a syringe pump kept at 37 °C, into the ESI source of a Bruker micrOTOF-QIII mass spectrometer (Bruker Daltonics, Coventry, UK) operating in the positive ion mode, and calibrated using ESI-L Low Concentration Tuning Mix (Agilent Technologies, San Diego, CA). MS data were acquired over the *m*/*z* range of 1500–3500 continuously for 5 min using Bruker oTOF Control software, with parameters as follows: dry gas flow 4 L min^−1^, nebuliser gas pressure 0.8 bar, dry gas 180 °C, capillary voltage 4000 V, offset 500 V, ion energy 5 eV, collision RF 600 Vpp, collision cell energy 10 eV.

Processing and analysis of MS experimental data were carried out using Compass DataAnalysis version 4.1 (Bruker Daltonik, Bremen, Germany). Neutral mass spectra were generated using the ESI Compass version 1.3 Maximum Entropy deconvolution algorithm over a mass range of 17 300–18 000 Da for the monomer and 34 850–35 810 Da for the dimer. Exact masses are reported from peak centroids representing the isotope average neutral mass. For apo proteins, these are derived from *m*/*z* spectra, for which peaks correspond to [*M* + *nH*]^*n*+^/*n*. For holo proteins, where the cluster contributes charge, peaks correspond to [*M* + (Fe–S)^*x*+^ + (*n* − *x*)*H*]^*n*+^/*n*, where *M* is the molecular mass of the protein, Fe–S is the mass of the particular iron–sulfur cluster of *x* + charge, *H* is the mass of the proton and *n* is the total charge. In the expression, the *x* + charge of the iron–sulfur cluster offsets the number of protons required to achieve the observed charge state (*n*+).^24^ Mass spectra are plotted as percentage relative abundances, where the most abundant species is arbitrarily set to 100% and all other species are reported relative to it.

### Electrophoretic mobility shift assays (EMSAs)

A DNA fragment (581 bp) from the *fhuA* promoter region containing the RirA IRO box consensus sequence TGACTAAAATAATCAT was PCR-amplified from *R. leguminosarum* genomic DNA with primers that contained a 6-carboxyfluorescin (FAM) modification at their 5′ end (Eurofins). Binding reactions in 20 μL were set up on ice in 1× binding buffer (10 mM Hepes, 50 mM NaCl, 2.5% sucrose, 2.5 mM MgCl_2_, 1 mM DTT, pH 7.9). DNA (20 nM) was incubated with varying concentrations of wild-type, N8C and N8D RirA, as indicated in figure legends. Then, 2 μL loading dye (50% (v/v) glycerol, 0.1% (w/v) bromophenol blue, 50% (v/v) 10× binding buffer) was added and samples were incubated on ice for 5 min. A 7.5% (v/v) polyacrylamide gel was pre-run for 2 min in TBE running buffer (45 mM Tris, 45 mM boric acid, 1 mM EDTA). Binding reactions were separated on the 7.5% (v/v) polyacrylamide gel at 30 mA for 45 min using a Mini Protean III System (Bio-Rad). Gels were visualised using an excitation wavelength of 473 nm on a GE Typhoon FLA 9000 Scanner (Cytiva). Apo proteins were prepared by mixing 5 μM of each cluster-containing protein with 5 mM EDTA overnight outside of the anaerobic cabinet, followed by buffer exchange. The lack of the [4Fe–4S] cluster was confirmed by UV-visible absorbance spectroscopy.

### Surface plasmon resonance (SPR)

A 30 bases-long oligonucleotide containing the RirA-binding IRO box consensus sequence of the *fhuA* promoter region was purchased from Integrated DNA Technologies or Eurofins Genomics, along with the reverse oligo, which also contained the ReDCaT Linker.^25^ Oligos were diluted to 100 μM in water and annealed at equal molarity by heating the solution to 95 °C for 10 min and allowing it to cool. Proteins were diluted in 10 mM Hepes, 150 mM NaCl, 0.05% (v/v) Tween, pH 7.4 to a final concentration of 0–125 nM dimeric RirA, as indicated in figure legends. The oligonucleotide samples were diluted to 200 nM in 10 mM Hepes, 150 mM NaCl, 0.05% (v/v) Tween, pH 7.4. Apo proteins were prepared as for EMSA experiments. [2Fe–2S] N8C RirA was prepared by mixing reconstituted [4Fe–4S] protein with aerobic binding buffer just before dilution to the final end concentration. SPR experiments were performed at 20 °C on a Biacore™ 8K system (Cytiva) using a multi cycle affinity method protocol. In each cycle, DNA was injected over a pre-prepared ReDCaT chip at 10 μL min^−1^ for 60 s to capture the probes. Test proteins were then injected for 60 s, followed by another 60 s of buffer, both at 50 μL min^−1^, to allow any interactions to equilibrate and/or dissociate. After measuring binding response, a solution of 1 M NaCl, 50 mM NaOH was flowed over the chip for 60 s at 10 μL min^−1^ to regenerate it for the following cycle. Experiments were performed in duplicate for each protein concentration, and two independent runs were performed. The analyte response at each concentration was averaged, and then normalised according to eqn (1):

1 Relative response = analyte response/*A*_max_

where the analyte response is the averaged response units (RU) at each concentration, and *A*_max_ is the maximum averaged analyte response in the titration. The relative response was then plotted against the dimeric protein concentration and fitted to a suitable binding equation. Use of an equation describing simple binding did not give a satisfactory fit for cluster-bound forms of wild-type and N8D RirA because of their sigmoidal shape. Thus, eqn (2) was employed:

2 Relative response = *Y*_max_(*x*^*n*^/(*K*_d_^*n*^ + *x*^*n*^))

where *Y*_max_ is the maximum relative binding response, *x* is the protein concentration, *K*_d_ is the binding affinity and *n* is the Hill coefficient. Eqn (2) allows for characterisation of cooperativity, which most likely results from binding of one RirA protomer affecting binding of the other (see Discussion). Fitting was performed in OriginPro, Version 2021b (OriginLab Corporation).

### *In vivo* methods

*E. coli* strain 803 and *R. leguminosarum* strains J251 (wild type) and J397 (*rirA*^*−*^ mutant) were grown routinely as previously described,^26^ and in iron-replete and iron-depleted media as described in ref. 2. N8C and N8D *rirA* derivatives with their native promoter sequence (123 bp upstream of the *rirA* ATG) were synthesised at Integrated DNA Technologies (Leuven, Belgium) and sub-cloned using *Eco*RI and *Nde*I into the vector pLMB509.^27^ The *rirA* gene was cloned in the opposing direction to the taurine inducible promoter in pLMB509. The sub-cloned N8C and N8D *rirA* plasmids and pBIO1306 (a *vbsC* promoter *lacZ* reporter clone^28^) were conjugated into *R. leguminosarum* strains separately using the helper plasmid pRK2013.^29^ The presence of plasmids within transconjugants was confirmed by PCR. β-Galactosidase assays and qualitative^30^ chrome azural sulfonate (CAS) tests for siderophore production were conducted as previously described.^30,31^

## Results

### N8D and N8C RirA variants have significantly different cluster environments

The identity, or indeed the existence, of a fourth cluster ligand in RirA is unknown. Phylogenetic analyses of the Rrf2 family revealed that the RirA clade is most closely related to the NsrR clade.^3,16^ Like RirA, NsrR homologues coordinate a [4Fe–4S] cluster,^4^ and the structure of *S. coelicolor* NsrR revealed that the fourth cluster ligand is an Asp residue from the other protomer of the dimer.^15^ The corresponding residue in RirA is an Asn that is highly conserved amongst RirA homologues (Fig. S2†). Three-dimensional modelling of RirA based on the structure of NsrR^15^ indicated that, compared to Asp8, Asn8 may be in a different rotamer, to prevent sub-optimal interactions of the amide side chain with either Arg12 or the cluster. Furthermore, the modelling indicated that the substitution of Asn8 for Asp may yield a structure of the N8D RirA variant that is very similar to that of NsrR, given the relatively few residue differences in the immediate cluster environment (Fig. 1). We were also interested in whether a conventional all-Cys ligand cluster coordination (such as that found in the O_2_-sensor FNR^32^) might be achievable in RirA. Modelling of a substitution of Asn8 for Cys indicated that a conformational change would be required for the Cys thiol to be able to coordinate the cluster. To test these proposals plasmids encoding N8D and N8C variants of RirA were generated, and the proteins purified for *in vitro* analysis.

**Fig. 2:**
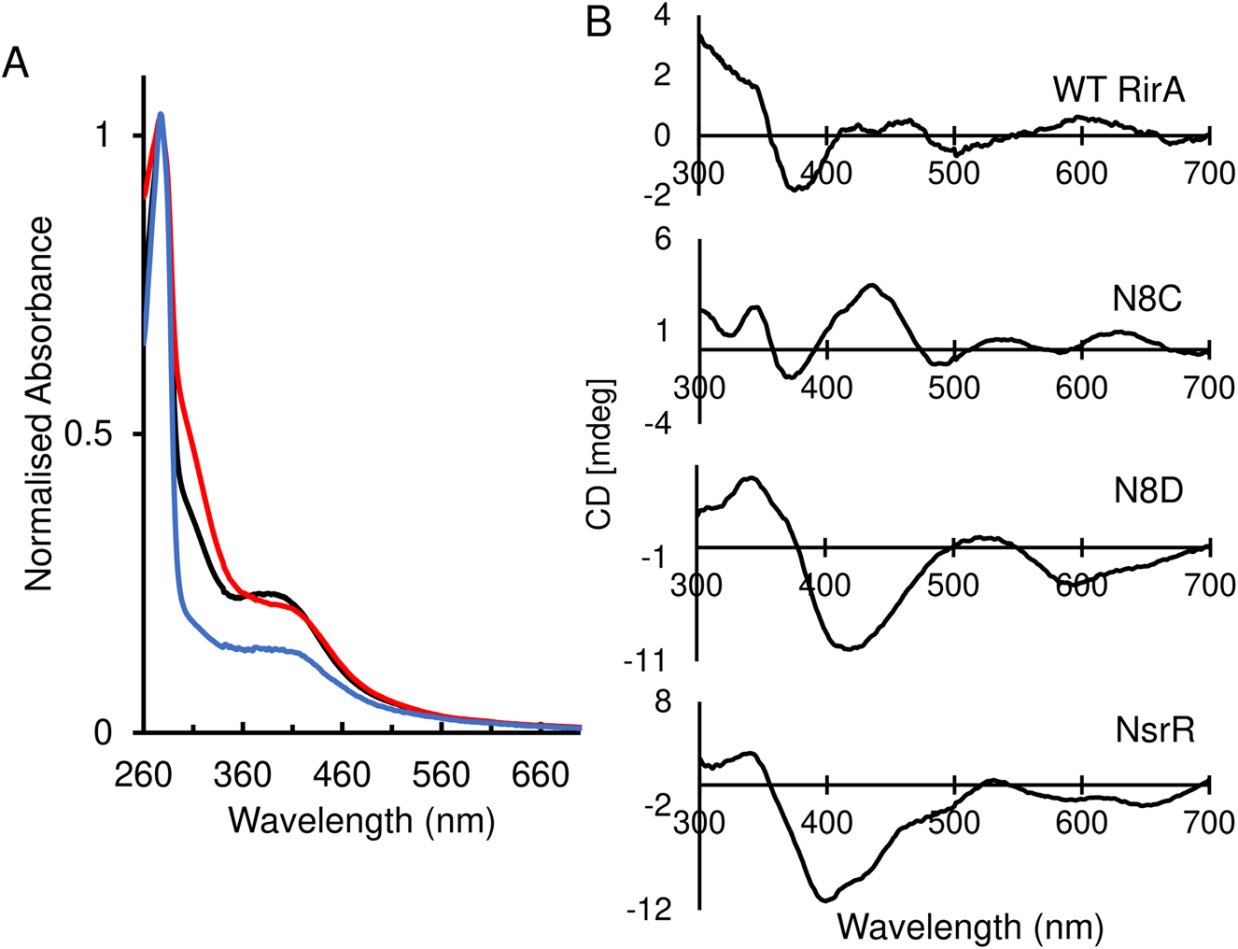
Spectroscopic characterisation of as-isolated RirA variants. (A) Normalised UV-visible spectra of reconstituted wild-type RirA (black line) and as-isolated variants N8D (red line) and N8C (blue line) RirA. (B) Circular dichroism (CD) spectra for reconstituted wild-type RirA, and as-isolated variants N8D and N8C RirA, as labelled. The spectrum of *S. coelicolor* NsrR^4^ is also shown for comparison. Samples were prepared in 25 mM Hepes, 50 mM NaCl, 750 mM KCl, pH 7.5. Spectra were recorded using a 1 cm pathlength anaerobic quartz cuvette.

Optical spectra of the N8D variant in its as-isolated form revealed a different cluster environment compared to wild-type RirA. The UV-visible absorbance maximum for the wild-type RirA cluster is at 382 nm, which is unusual for a [4Fe–4S] cluster in that it is below 400 nm.^33^ In the spectrum of N8D RirA, the cluster maximum shifted to 410 nm (Fig. 2A), a more conventional maximum for a [4Fe–4S] cluster, and one that closely resembles that of NsrR (maximum at 406 nm).^4^ CD spectroscopy revealed a strikingly different spectrum for N8D RirA compared to wild-type RirA: the negatively signed band below 400 nm was significantly more intense and shifted to ∼410 nm, and the sign of the low intensity positive band at 600 nm reversed (Fig. 2B). Thus, both absorbance and CD spectra of N8D RirA closely resembled those of NsrR^4^ (Fig. 2).

The UV-visible absorbance spectrum of the N8C variant was very similar to that of wild-type RirA (Fig. 2A). The CD spectrum revealed that the two low intensity bands between 400–500 nm were replaced by a more intense single broad band in the same region, and the low intensity positive band at 600 nm was shifted to ∼620 nm (Fig. 2B). Thus, smaller changes in optical properties were detected for N8C RirA compared to N8D RirA.

### N8D and N8C substitutions affect the stability of [4Fe–4S] RirA

**Fig. 3:**
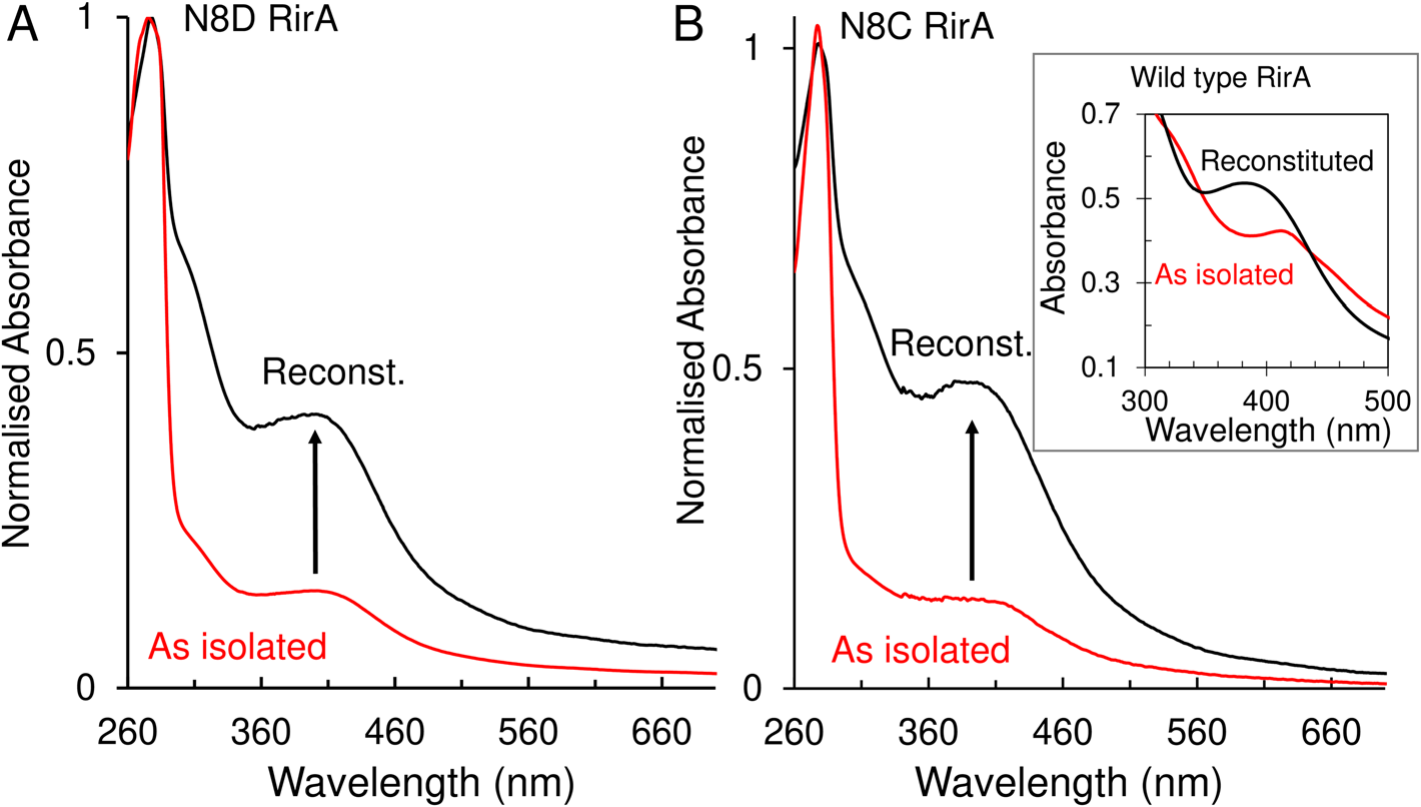
UV-visible absorbance characterisation of Asn8 variants of RirA following purification and *in vitro* cluster reconstitution. (A) Normalised UV-visible absorbance spectra for comparison between as isolated N8D RirA (red line) and reconstituted N8D RirA (black line). Arrow indicates increase of maximum peak at 410 nm corresponding to an increase in [4Fe–4S] cluster loading. (B) As in (A) but for N8C RirA. Inset, UV-visible absorbance spectra of as-isolated (red line) and reconstituted (black line) wild-type RirA for comparison, adapted from ref. 8. Spectra were recorded for as-isolated, and reconstituted Asn8 variant RirA proteins in 25 mM Hepes, 2.5 mM CaCl_2_, 50 mM NaCl, 750 mM KCl, pH 7.5. Measurements were obtained using a 1 mm sealed anaerobic cuvette.

A major and functionally important characteristic of the wild-type RirA [4Fe–4S] cluster is its fragility, through which low iron levels and O_2_ are sensed.^7,8^ The stabilities of the N8D and N8C clusters were therefore investigated. *In vitro* reconstitution resulted in 75–100% cluster loading of the RirA variants, and UV-visible absorbance and CD spectral characteristics were, in both cases, very similar to those of the as isolated protein (Fig. 3 and S3†). Conversely, as isolated and reconstituted wild-type RirA gave rise to different spectra, due to the breakdown of the cluster during the purification procedure (see inset Fig. 3B).^8^

**Fig. 4:**
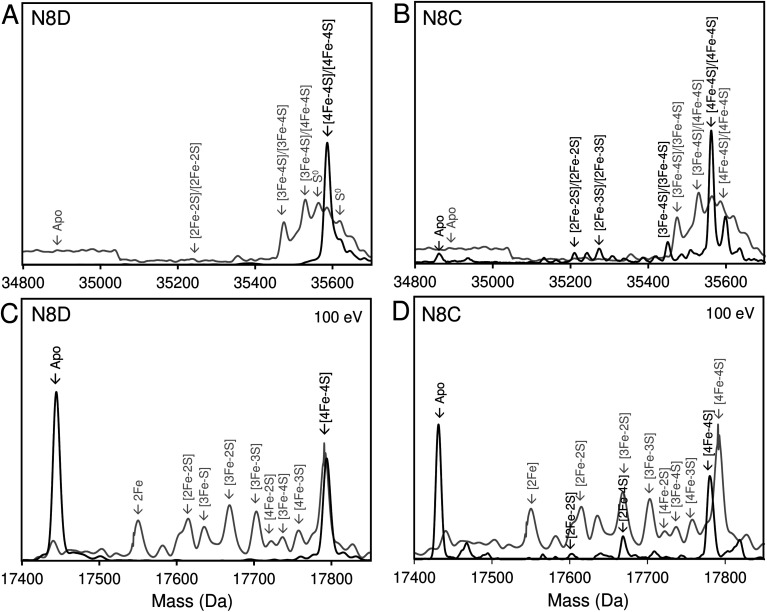
Native ESI-MS characterisation of reconstituted N8D and N8C RirA in comparison with wild-type protein. (A) Deconvoluted mass spectrum of dimeric N8D RirA showing the only significant species, namely, dimeric, [4Fe–4S]/[4Fe–4S] N8D RirA with no observable cluster degradation species. (B) Deconvoluted mass spectrum of dimeric N8C RirA showing that the [4Fe–4S]/[4Fe–4S] form is the major species, with low intensity peaks due to [3Fe–4S]/[3Fe–4S], [2Fe–2S]/[2Fe–2S] (and persulfidated forms), and apo N8C RirA also present. The deconvoluted mass spectrum of dimeric wild-type RirA is shown for comparison in grey^7^ in (A) and (B). (C) Deconvoluted mass spectrum of the monomeric region of N8D RirA when 100 eV isCID was applied (from *m*/*z* 1500 to 2200) showing apo and [4Fe–4S] N8D RirA, with no cluster degradation species in between. (D) Deconvoluted mass spectrum of the monomeric region of N8C RirA when 100 eV isCID was applied (from *m*/*z* 1500 to 2200) showing apo, [2Fe–2S], [2Fe–4S] and [4Fe–4S] N8C RirA. The deconvoluted mass spectrum of the monomeric region of wild-type RirA is shown for comparison in grey, with cluster degradation species labelled.^7^ N8D and N8C RirA proteins (28 μM in cluster following reconstitution) were exchanged into 250 mM ammonium acetate, pH 7.3.

Native (non-denaturing) ESI-MS was performed for reconstituted N8D and N8C RirA proteins under the same conditions previously employed for wild-type RirA.^7,8^ For the latter, the *m*/*z* spectrum contained peaks in two distinct regions upon ionization, corresponding to monomeric (600–1500 *m*/*z*), and dimeric (1800–3000 *m*/*z*) forms. In contrast, monomeric forms of N8D and N8C RirA proteins were not observed, with the *m*/*z* spectrum corresponding only to the dimeric region (Fig. S4A, B,†4A and B). Deconvolution revealed major peaks at 35 585 Da and 35 561 Da, corresponding to the [4Fe–4S]/[4Fe–4S] forms of N8D and N8C RirA dimers, respectively (Fig. 4A and B). Importantly, for N8D RirA, this was the only protein-associated Fe–S species observed. This differs significantly from the deconvoluted spectrum of wild-type RirA (see spectra in grey, Fig. 4), which revealed a whole range of RirA-associated cluster breakdown products, including [3Fe–4S]/[4Fe–4S], [3Fe–4S]/[3Fe–4S], [3Fe–3S]/[3Fe–3S], and [2Fe–2S]/[2Fe–2S], in addition to the intact [4Fe–4S]/[4Fe–4S] RirA dimer.^7,8^ For N8C RirA, minor peaks at 35 450 and 35 210 Da were also observed, which correspond to the [3Fe–4S]/[3Fe–4S] and [2Fe–2S]/[2Fe–2S] N8C dimers, as well as multiple sulfur adducts of the latter.

Both N8D and N8C RirA dimers could be disturbed by in-source collision-induced dissociation (isCID, up to 100 eV) during ESI-MS, whereby collisions between inert gas and analyte protein molecules impart energy to the protein, resulting in the loss of weaker non-covalent interactions. This resulted in the emergence of peaks in a spectral region corresponding to the monomer (Fig. 4C, D, S4C and D†), and also in cluster degradation products. Deconvolution of the monomer region revealed two significant peaks corresponding to [4Fe–4S] and apo forms of N8D RirA (Fig. 4C), and to [4Fe–4S], [2Fe–2S] and apo forms of N8C RirA (Fig. 4D). These data showed that substitution of Asn8 significantly enhanced cluster stability, with the N8D version of RirA being especially resilient to decomposition.

### N8D and N8C substitutions in RirA affect iron-sensing capabilities

Low-iron conditions, mimicked *in vitro* by the presence of a chelator such as EDTA, were previously shown to result in cluster conversion/degradation in wild-type RirA.^7,8^ Considering the observations above about cluster stability, the effects of low iron on the N8D and N8C RirA clusters were followed using both UV-visible absorbance spectroscopy and native ESI-MS.

For UV-visible absorbance experiments, wild-type, N8D and N8C RirA proteins were exposed to 1 mM EDTA, under aerobic and anaerobic conditions, and spectral changes were followed as a function of time. For wild-type RirA, the absorbance maximum peak shifted from 382 nm to 425 nm,^8^ and by plotting the difference between absorbance at these wavelengths (*A*_382 nm_ − *A*_425 nm_), the cluster degradation could be followed (Fig. S5†). This showed that, under anaerobic conditions, intensity due to the wild-type RirA [4Fe–4S] cluster was lost gradually over several hours in response to low iron, with the formation of a relatively stable [2Fe–2S] cluster intermediate. Fitting of the kinetic data (Δ*A*_382–425 nm_) with a single exponential function gave a rate constant of ∼0.009 min^−1^ (Fig. S5†). The combined exposure to low iron and O_2_ resulted in a very similar cluster conversion reaction, except that it occurred significantly more rapidly, with a rate constant 3-fold higher (∼0.029 min^−1^) than for the same reaction under anaerobic conditions (Fig. S5†).

**Fig. 5:**
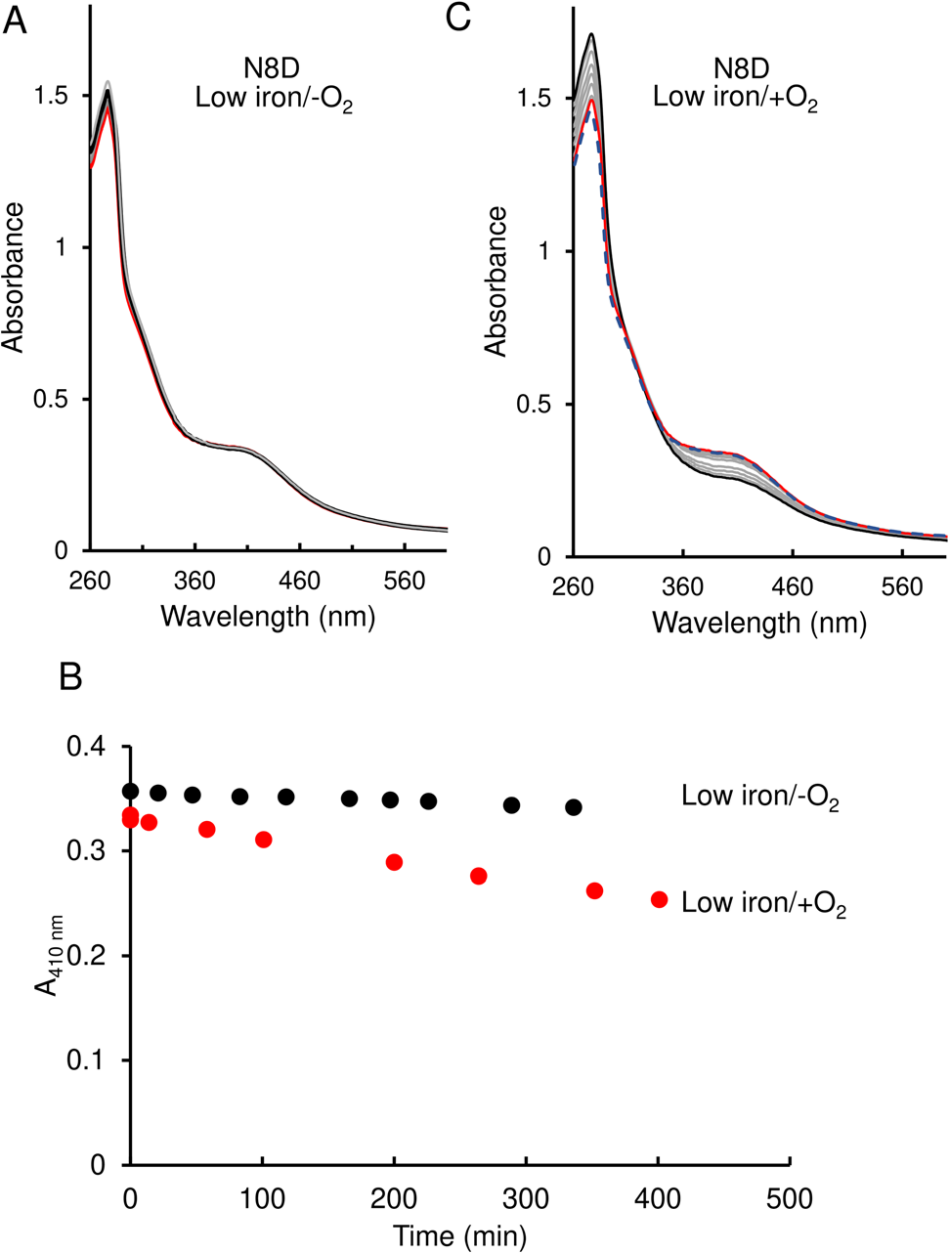
Effect of 1 mM EDTA on N8D RirA [4Fe–4S] cluster. (A) UV-visible absorbance spectra of N8D RirA recorded over several hours following the addition of 1 mM EDTA, under anaerobic conditions. The initial spectrum is in red, all intervening spectra are in grey, end point spectrum is in black (B) Plot of absorbance change obtained from anaerobic (black circles) and aerobic (red circles) experiments: *A*_410 nm_ was plotted as a function of time. (C) As in (A) but under aerobic conditions. The initial spectrum prior to O_2_ exposure is a blue dashed line, the initial spectrum following exposure to O_2_ is shown in red, while all intervening spectra are in grey, and the end point spectrum is in black. For both experiments, N8D RirA (29 μM in cluster, as isolated) was in 25 mM Hepes, 2.5 mM CaCl_2_ 50 mM NaCl, 750 mM KCl, pH 7.5. Measurements were obtained using a 1 cm pathlength sealed anaerobic quartz cuvette.

In contrast to the wild-type protein, under low-iron, anaerobic conditions the UV-visible absorbance spectrum for N8D RirA remained largely unchanged over the 400 min of measurement, indicating that the cluster has little or no sensitivity to low iron (Fig. 5A and B). Furthermore, under aerobic conditions, although a drop in absorbance was observed, this occurred slowly, with the *A*_410 nm_ decreasing by only ∼20% over 350 min, and no shift in the peak maximum was observed (Fig. 5B and C).

**Fig. 6:**
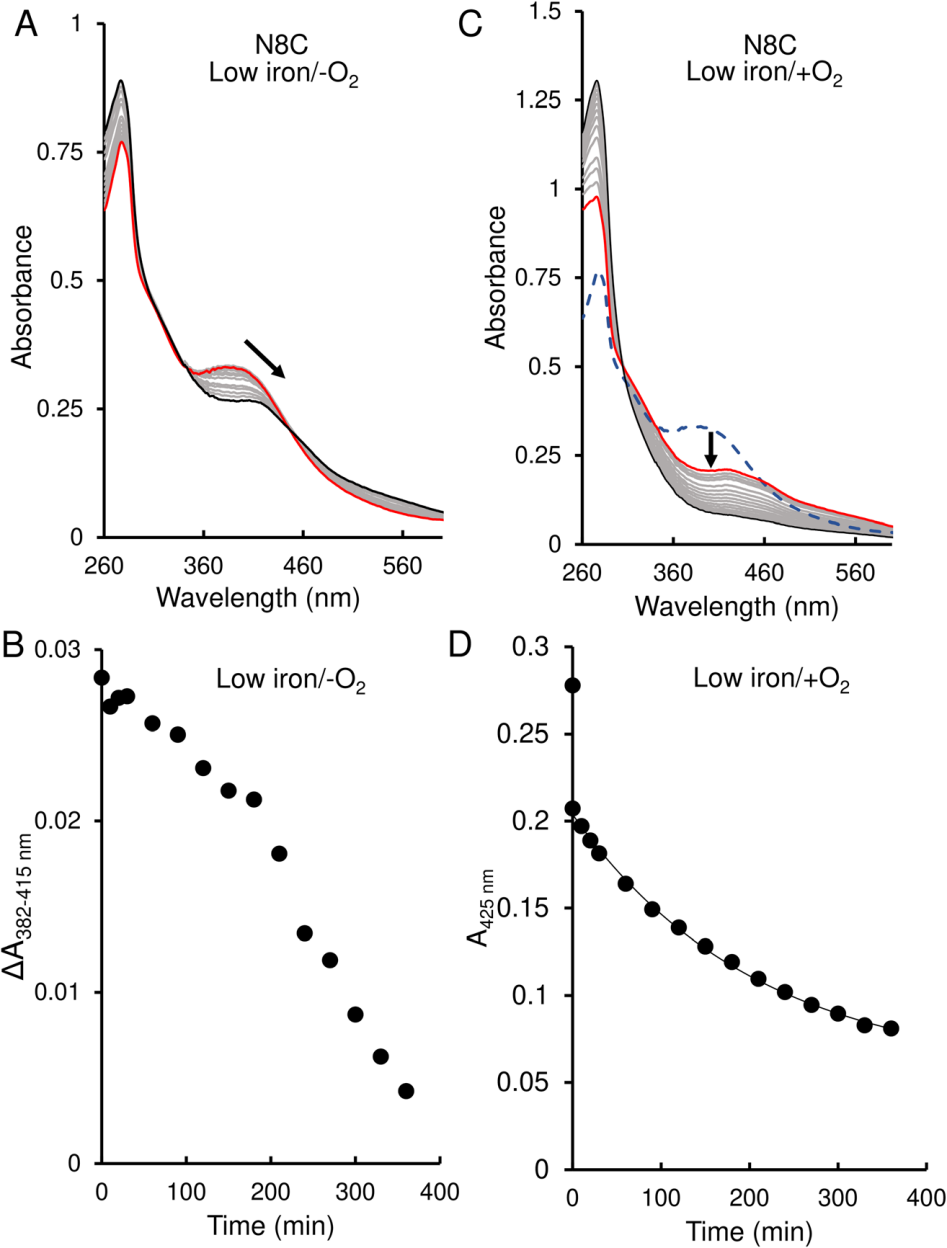
Effect of 1 mM EDTA on N8C RirA [4Fe–4S] cluster. (A) UV-visible absorbance spectra of N8C RirA recorded over several hours following the addition of 1 mM EDTA, under anaerobic conditions. (B) Plot of absorbance change obtained from anaerobic experiments: *A*_382 nm_ − *A*_415 nm_ plotted as a function of time. (C) As in (A) but under aerobic conditions. The initial spectrum prior to O_2_ exposure is a blue dashed line, the initial spectrum following exposure to O_2_ is shown in red, while all intervening spectra are in grey, and the end point spectrum is in black. (D) As in (B) but for aerobic experiment and with absorbance *A*_415 nm_ plotted as a function of time. The immediate decrease in absorbance upon exposure to O_2_ reflects conversion from a [4Fe–4S] to a [2Fe–2S] cluster. The post-O_2_ exposure data points were fitted using a single exponential. For both experiments, N8C RirA (29 μM in cluster following reconstitution) was in 25 mM Hepes, 2.5 mM CaCl_2_ 50 mM NaCl, 750 mM KCl, pH 7.5. Measurements were obtained using a 1 cm pathlength sealed anaerobic quartz cuvette. Arrows indicate direction of change.

The response of N8C RirA demonstrated some similarities to that of the wild-type protein. In the presence of EDTA, under anaerobic conditions, UV-visible absorbance spectra revealed a shift in the peak maximum from 382 nm to 415 nm (Fig. 6A), similar to the shift observed for the wild-type protein. A plot of the absorbance difference between the peak maxima (Δ*A*_382–415 nm_) (Fig. 6B), indicated that under anaerobic conditions, and in response to low iron, intensity due to the N8C [4Fe–4S] cluster was lost gradually over several hours during which the [2Fe–2S] cluster form accumulated. After around 200 min, the Δ*A*_382–415 nm_ decreased more rapidly, indicating degradation of the [2Fe–2S] cluster. This was somewhat slower than that for the wild-type protein, as the decay had yet to plateau after 360 min.

In contrast, under aerobic conditions, the absorbance peak maximum instantly shifted to 415 nm (Fig. 6C), suggesting that the N8C [4Fe–4S] cluster very rapidly converts to the [2Fe–2S] form in the presence of O_2_. This cluster form subsequently decayed further, as indicated by the decrease in absorbance at that wavelength (Fig. 6D), which, when fitted to a single exponential function, gave a rate constant of 0.0049 min^−1^, ∼6-fold lower than the equivalent for wild-type RirA.

**Fig. 7:**
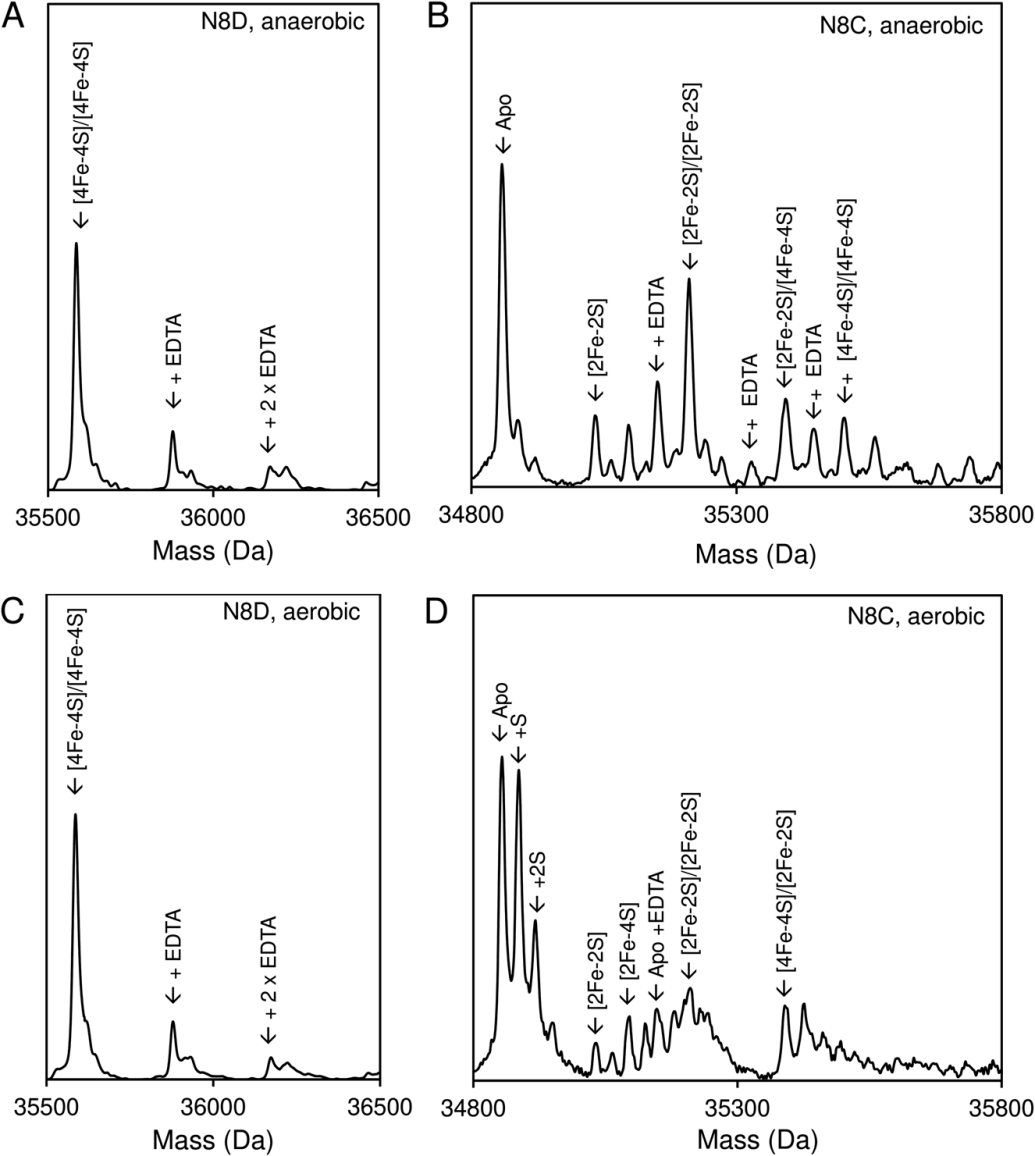
Effect of low iron conditions on N8D and N8C RirA. (A) Deconvoluted final mass spectrum of N8D RirA 30 min after exposure to EDTA in the absence of O_2_. (B) Deconvoluted final mass spectrum of N8C RirA 30 min after exposure to EDTA in the absence of O_2_. (C) As in (A) but in the presence of 230 μM O_2_. (D) As in (B) but in the presence of 230 μM O_2_. In all cases, spectra show mass peaks corresponding primarily to [4Fe–4S]/[4Fe–4S] dimeric N8D and N8C RirA, with any EDTA adducts as indicated. For N8C, other FeS protein-associated peaks are assigned. Both N8C and N8D RirA (28 μM in cluster following reconstitution) were exposed to 250 μM EDTA at 37 °C in 250 mM ammonium acetate, pH 7.3.

Native ESI-MS experiments were also performed following exposure to EDTA under both aerobic and anaerobic conditions, as previously reported for wild-type RirA.^7^ After a 30 min exposure to EDTA, under either anaerobic or aerobic conditions, N8D RirA remained unchanged as a [4Fe–4S]/[4Fe–4S] dimer with no monomerisation, or cluster breakdown occurring (Fig. 7A and C). This is in contrast to wild-type RirA where no [4Fe–4S] cluster remained after 30 min under either aerobic or anaerobic conditions.^7^ Together with the UV-visible absorbance kinetic experiments described above, the data demonstrated that N8D RirA does not sense low iron and does not undergo the cluster conversion previously observed for wild-type RirA.

In contrast, the N8C version showed much greater instability; after a 30 min anaerobic exposure to EDTA, the dimeric apo protein was the major species present (Fig. 7B). Some protein-associated FeS species, in addition to the [4Fe–4S]/[4Fe–4S] form, were also present, in particular the [2Fe–2S]/[2Fe–2S] form, as suggested by UV-visible absorbance spectra. However, the extent of degradation at 30 min was less than that observed for wild-type RirA.^7^ Under aerobic conditions, a 30 min exposure to EDTA resulted mainly in apo protein with less of the [2Fe–2S]/[2Fe–2S] form and little [4Fe–4S] cluster remaining (Fig. 7D). This is consistent with the UV-visible absorbance data that showed cluster degradation to occur more rapidly under aerobic conditions. Overall, the [4Fe–4S] cluster of N8C RirA is susceptible to degradation, resulting in both a partially stable [2Fe–2S] form and apo proteins. Thus, it appears to retain some capacity to sense iron, but is also significantly more stable than the wild-type protein.

### Asn8 variants of RirA retain *in vitro* DNA-binding capability

Wild-type [4Fe–4S] RirA represses genes involved in iron homeostasis and acquisition under iron-replete conditions by binding IRO boxes,^1^ and the degradation of the [4Fe–4S] cluster causes a loss of DNA binding, with the intermediate [2Fe–2S] form having a reduced affinity for IRO boxes.^8^ Here, the DNA binding properties of the stabilised [4Fe–4S] N8D and N8C RirA variants were investigated.

**Fig. 8:**
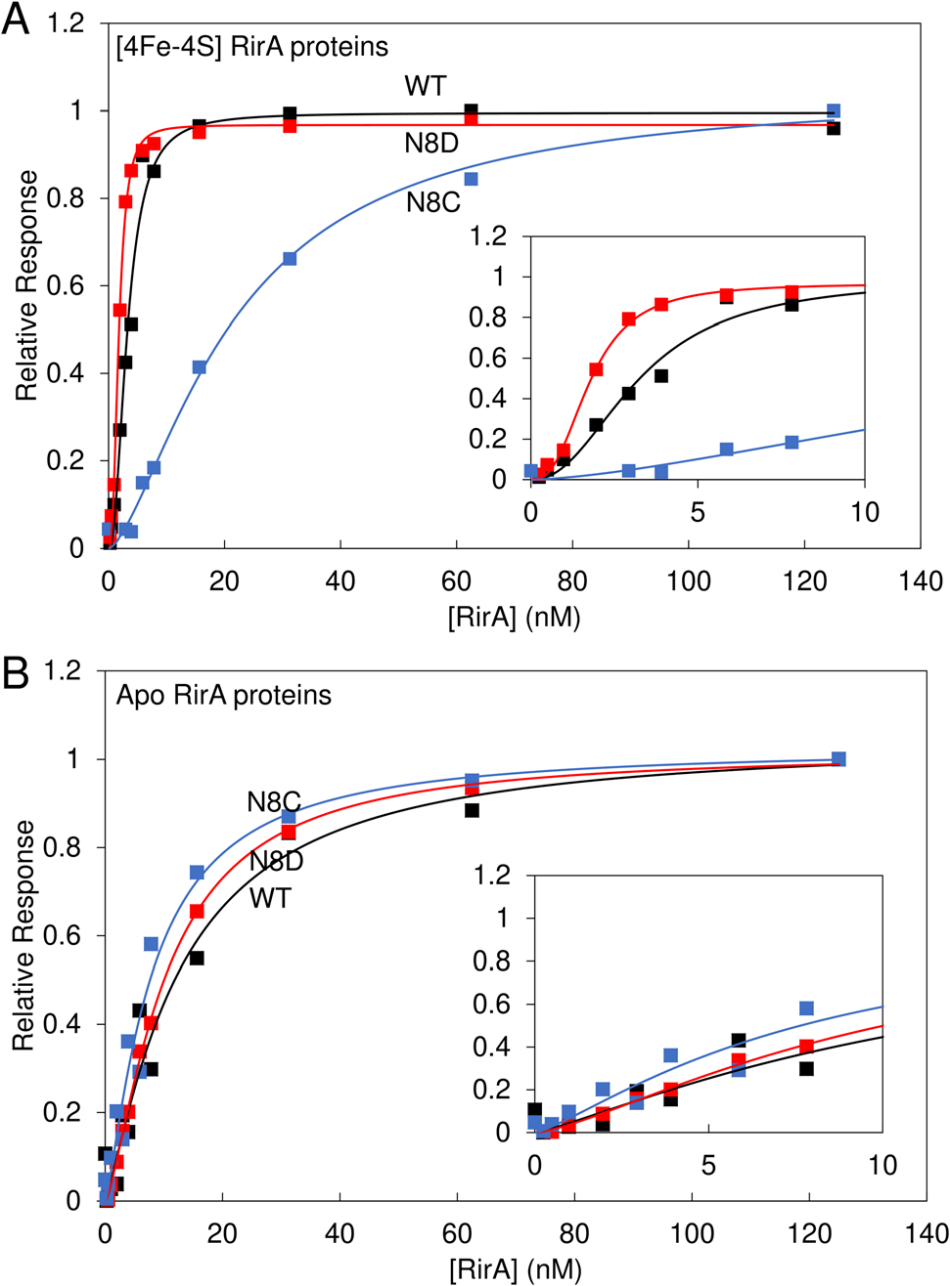
Binding of wild-type, N8D and N8C RirA to the *fhuA* promoter probed by SPR. Analyte binding response of wild-type (black), N8D (red) and N8C (blue) RirA to 200 nM 30 bp *fhuA* promoter region containing the IRO box consensus sequence in the (A) reconstituted [4Fe–4S] and (B) apo forms. Insets show the early part of the titrations in more detail. Clear sigmoidal behaviour was observed for wild-type and N8D [4Fe–4S] RirA proteins, indicating cooperativity, and the Hill equation was used for fitting, see Table 1. For the [4Fe–4S]-bound form, 0–125 nM dimeric protein concentration was incubated with 200 nM of the 30 bp *fhuA* oligo in 10 mM Hepes, 150 mM NaCl, 0.05% Tween, pH 7.4. Apo proteins were prepared in 5 mM EDTA overnight, and then titrations were performed as for holo proteins.

Electrophoretic mobility shift assays (EMSAs) demonstrated that the [4Fe–4S] forms of both N8D and N8C RirA are still able to bind to a 500 bp DNA fragment containing the RirA-regulated *fhuA* promoter and its IRO box.^1^ Indeed, the N8D and N8C RirA proteins bound with higher and lower affinity than wild-type RirA, respectively (Fig. S6†). To gain more detailed insight into the apparent differences in affinity for the *fhuA* promoter observed by EMSAs, surface plasmon resonance (SPR) was utilised (Fig. 8). SPR enables high-sensitivity measurements of the binding of analyte species (here, RirA proteins) to an immobilised ligand (*fhuA* IRO box DNA), yielding binding affinities,^34–36^ and overcomes the main problems associated with determining DNA-binding affinities of Fe–S cluster-containing regulatory proteins by EMSA (see Fig. S6†). For each of the variants and the wild-type protein, the response of binding in the holo and apo forms was normalised. For the holo proteins, satisfactory fits of the binding data could not be obtained using a simple binding equation (see Fig. S7†), because the plots are sigmoidal (inset Fig. 8A), indicating that binding is cooperative.

Use of the Hill equation gave satisfactory fits, with *K*_d_ values of ∼3 nM for wild type, ∼2 nM for N8D RirA and ∼20 nM for N8C (see Table 1). These values are qualitatively consistent with the EMSA data (Fig. S6†), with N8C binding significantly weaker than that of N8D and wild-type RirA. The shape of the plots and Hill coefficients obtained from the fits (Table 1) clearly indicated that wild-type and N8D RirA proteins bind in a positively cooperative manner. N8C RirA also exhibited some evidence of positive cooperativity, but this was much less clear than for wild-type and N8D RirA. Equivalent SPR and EMSA experiments with apo proteins revealed weaker binding to the *fhuA* IRO box than by the respective holo proteins (Fig. 8B and S8†), with fitting of the SPR data giving *K*_d_ values in the range of 8–12 nM for the three (Table 1), and with no clear evidence for cooperativity (Fig. 8B, Table 1).

**Table 1:** Binding parameters derived from SPR measurements of RirA protein binding to DNA containing the RirA-regulated *fhuA* promoter sequence

Protein	*K*_d_ (nM)	Hill coefficient
**[4Fe–4S] form**		
Wild-type RirA	3.2 ± 0.2	2.2 ± 0.3
N8D RirA	1.8 ± 0.1	2.7 ± 0.2
N8C RirA	21.7 ± 2.1	1.5 ± 0.1

**[2Fe–2S] form**		
N8C RirA	10.2 ± 1.3	1.6 ± 0.2

**Cluster-free (apo) form**		
Wild-type RirA	12.8 ± 3.2	1.2 ± 0.2
N8D RirA	10.3 ± 0.9	1.4 ± 0.3
N8C RirA	8.0 ± 1.3	1.3 ± 0.2

Data for N8C RirA from both SPR (Fig. 8) and EMSA (Fig. S6 and S7†) indicated that the affinity of the apo protein (Table 1) appears to be higher than that of the [4Fe–4S] cluster form, suggesting that the presence of the cluster disfavours DNA binding in this variant. As UV-visible spectroscopy and native ESI mass spectrometry indicated that N8C can also exist in the [2Fe–2S] form, and indeed appeared to rapidly convert to this form upon exposure to O_2_, the affinity of [2Fe–2S] N8C RirA for DNA was also investigated by SPR (Fig. S9†). This gave a *K*_d_ of ∼10 nM, with a Hill coefficient indicative of some positive cooperativity (Table 1). The measured affinity of the [2Fe–2S] form was similar to that of the apo protein, consistent with an at least partial decoupling of the status of the cluster and DNA binding in this variant.

### Effect of the N8D and N8C substitutions on regulatory properties of RirA *in vivo*

**Fig. 9:**
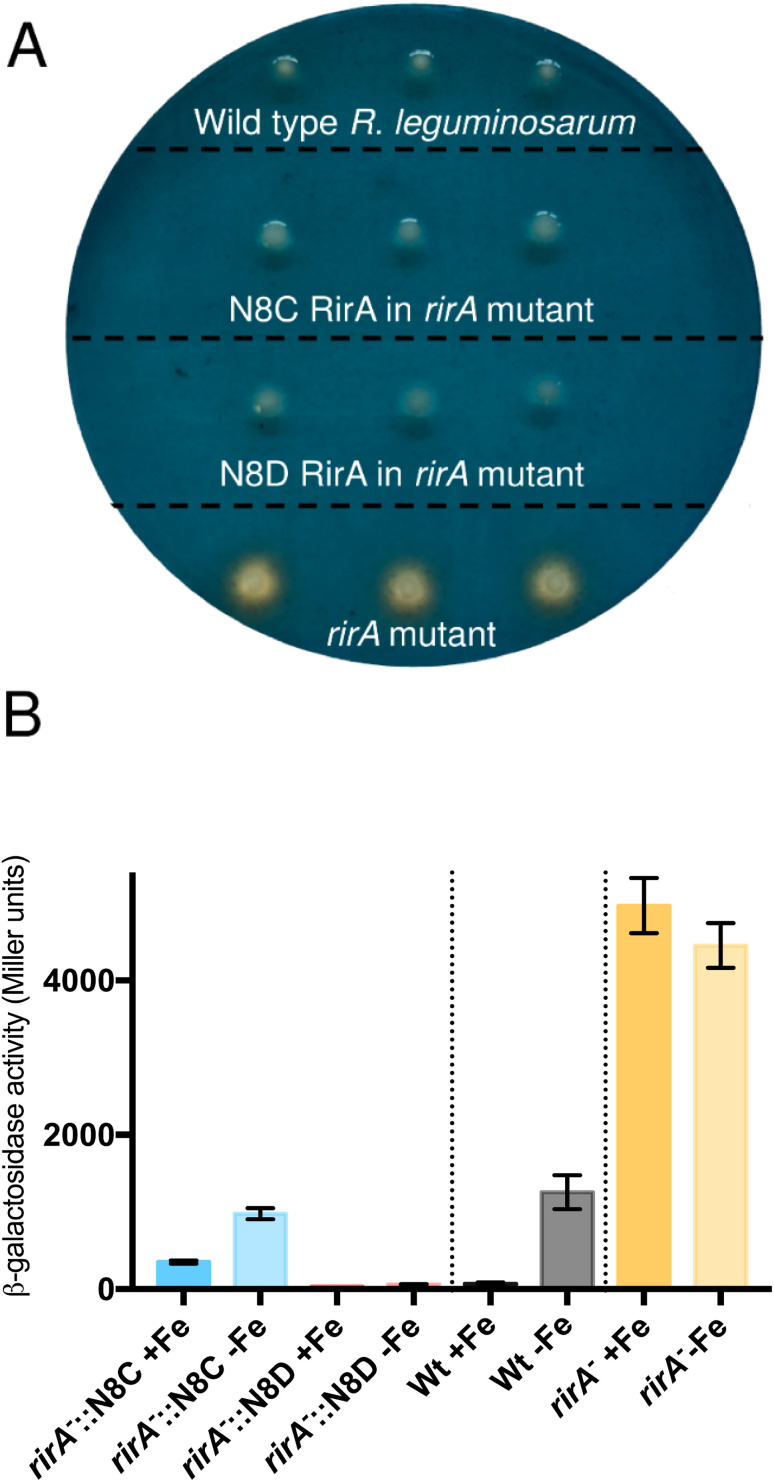
Effect of N8C and N8D versions of RirA on Fe-dependent expression of a *vbsC*–*lacZ* fusion. (A) Chrome azurol S (CAS) plate assays of siderophore production in wild-type *R. leguminosarum*, the *rirA*^*−*^ mutant, and the *rirA*^−^ mutant complemented by cloned *rirA* encoding Asn8 variants, as indicated. (B) β-Galactosidase activity (Miller units) resulting from the *vbsC* promotor *lacZ* fusion in wild-type *R. leguminosarum* (wild type, Wt, black), a *rirA*^*−*^ mutant (*rirA*^*−*^, orange) and a *rirA*^*−*^ mutant containing cloned N8C *rirA* or N8D *rirA* (blue and red, respectively). Cells were grown with Fe (+Fe), or without added Fe but in the presence of 20 mM 2,2′-dipyridyl (−Fe).

The surprising ability of [4Fe–4S] N8D RirA to bind DNA with affinity similar to (or even greater than) wild-type RirA, and for the N8C variant to also bind, albeit more weakly in the cluster-bound form, prompted the question of whether substitutions of Asn8 affect the *in vivo* function of RirA. To address this, a *R. leguminosarum rirA*^−^ mutant was employed. Growth on CAS-agar plates resulted in a characteristic larger orange halo around the *rirA*^−^ mutant compared to the wild-type strain, due to the production and export of the siderophore vicibactin that complexes iron from the blue Fe^3+^–CAS complex (Fig. 9A). Interestingly, genetic complementation of the *rirA*^−^ mutant with plasmids encoding N8D and N8C RirA proteins resulted in wild-type CAS phenotypes, where no orange-coloured halos around the colonies were visible. This indicated that iron uptake, and therefore vicibactin production, was no longer deregulated. These data support both N8D and N8C RirA proteins being able to bind IRO box sequences and repress transcription of Fe-responsive genes *in vivo*.

### N8D and N8C RirA variants show significantly different gene regulatory function

To further test whether N8D and N8C RirA proteins are still effective iron-responsive regulators *in vivo*, we investigated their ability to regulate the transcription of *vbsC*, which encodes an acetylase involved in the production of vicibactin^28^ and is repressed by RirA in iron-replete conditions.^2^ For this we utilised a *lacZ* fusion plasmid, pBIO1306, which reports *vbsC* transcription as β-galactosidase activity^28^ in wild-type and *rirA*^*−*^ mutant *R. leguminosarum* strains. As expected, *vbsC* transcription was Fe-repressed in wild-type *R. leguminosarum* but was expressed constitutively in a *rirA*^*−*^ mutant background, showing elevated β-galactosidase activity in both iron-replete and iron-depleted conditions (Fig. 9B). When cloned N8D and N8C forms of *rirA* were introduced into the *rirA*^*−*^ mutant, β-galactosidase activity was significantly lowered under both iron-replete and iron-depleted conditions. This is consistent with both the N8D and N8C RirA proteins binding the IRO box sequence in the *vbsC* promoter and functioning as repressors. However, neither N8D nor N8C RirA proteins fully restored the iron-responsive β-galactosidase activities observed for wild-type RirA, and both proteins were less effective iron-responsive regulators than the wild-type protein. N8C RirA-mediated repression of *vbsC* under iron-replete conditions was apparent, but the overall de-repression observed between iron-replete and iron-depleted conditions was ∼7-fold less than with the wild-type RirA protein. In contrast, the N8D RirA protein completely lost the ability to sense iron, since very low *vbsC* transcription levels were observed under both iron-replete and iron-depleted conditions. This is consistent with the *in vitro* stabilisation of the N8D [4Fe–4S] cluster described above.

## Discussion

RirA senses and responds to levels of intracellular iron *via* an unusual mechanism involving direct sensing through its iron–sulfur cluster. Previously, a range of biophysical approaches showed that RirA contains a fragile [4Fe–4S] cluster, and that this form of the protein binds cognate DNA sequences, repressing transcription of genes involved in iron uptake.^2,7,8,37^ This RirA cluster fragility provides the basis of iron-sensing, based on the following equilibrium: [4Fe–4S]^2+^ ⇌ [3Fe–4S]^0^ + Fe^2+^. Under iron-replete conditions, the [4Fe–4S] form is stable, but when iron is low, the equilibrium shifts towards the [3Fe–4S] form, which is unstable and degrades to a [2Fe–2S] cluster and then to apo RirA.^8^ To be an effective sensor of iron levels, the dissociation constant (*K*_d_) of the fourth, labile iron must be in the range of the free (chelatable) iron concentration within the cytoplasm. Although free iron levels are not known for *Rhizobium*, based on reported free iron levels in the *E. coli* cytoplasm,^38^ a *K*_d_ of ∼3 μM (ref. 7) fits well with iron-sensing capabilities.

The dissociation/re-association of the Fe^2+^ ion in the above equilibrium occurs with rates that are similar under both aerobic and anaerobic conditions, suggesting that, although RirA has both iron- and O_2_-sensing capabilities, primarily it is an iron sensor.^7^ O_2_ sensing occurs *via* the oxidation of the [3Fe–4S]^0^ intermediate to [3Fe–4S]^1+^, which decays more rapidly than the reduced form.^7^

It is clear that the lability of the fourth iron of the RirA [4Fe–4S] cluster is crucial for its function as an iron sensor, and that the facile loss of an iron ion under low iron conditions is not a feature shared by most [4Fe–4S] cluster proteins.^8^ This led us to hypothesise^7^ that the cluster is bound to RirA only by the three conserved Cys amino acid side chains, with the fourth, labile iron coordinated by a non-protein ligand such as water or hydroxide, as in aconitase.^39,40^ We further hypothesised that, if this was the case, it might be possible to introduce a fourth amino acid ligand, without significantly disrupting the RirA structure/conformation around the cluster. Based on the close phylogenetic relationship between RirA and NsrR clades,^3^ and the sequence similarities between RirA and the previously structurally characterised NsrR, in which the fourth ligand is an Asp residue (Asp8),^15^ the Asn residue at position 8 was replaced by Asp generating N8D RirA. Asn8 was also substituted by Cys, generating N8C RirA, to test if a four Cys residue coordination could be achieved.

Spectroscopic characterisation of the RirA variants showed that N8D RirA exhibited a cluster absorption maximum above 400 nm, which is characteristic of most [4Fe–4S] clusters, with overall optical properties strikingly similar to those of NsrR.^4^ Furthermore, in contrast to wild-type RirA, in which the [4Fe–4S] cluster undergoes conversion to a [2Fe–2S] cluster upon exposure to low iron/anaerobic conditions, N8D RirA was shown to have lost its sensitivity to low iron, in that it remained stable under these conditions. Under aerobic conditions, N8D RirA did undergo a minor degree of cluster degradation, but this was distinct from that observed for wild-type RirA, being very slow and not involving conversion to a transient [2Fe–2S] cluster intermediate. Additionally, native ESI MS did not show the presence of any protein-associated [3Fe–4S] species, which are crucial for the iron-sensing abilities of wild-type RirA.

*In vitro* data showed that the N8D RirA variant was still able to bind to the IRO box consensus sequence with a nanomolar affinity, similar to that of wild-type RirA. Thus, the introduction of Asp at position 8 did not cause a significant structural perturbation of the [4Fe–4S] cluster. In structurally characterised Fe–S proteins of the Rrf2 family, the cluster is important for the conformation of the DNA-binding domain.^6,15,41^ The apo forms of wild-type and N8D RirA exhibited, respectively, a 3- to 5-fold and 5- to 6-fold decrease in affinity (compared to their holo forms) for the RirA IRO box, indicating that the N8D substitution also did not significantly affect the conformational changes that occur upon loss of the cluster, which are transduced to the DNA-binding domain. The affinity of apo RirA remained relatively high, suggesting that this could be physiologically relevant. The difference in expression is consistent with apo RirA retaining DNA-binding capability.

Hill coefficients obtained from the fitting of SPR data for binding of [4Fe–4S] forms of wild-type and N8D RirA implied cooperative binding of these proteins to cognate DNA. This behaviour was not exhibited by their apo forms, suggesting that the observed cooperativity could be physiologically relevant. However, the nature of any cooperativity is unclear, because structures of Rrf2 family regulators bound to DNA reveal the binding of a single protein dimer to each DNA molecule.^18,41,42^ It is noteworthy that IscR has also been shown to exhibit cooperative DNA binding at type 2 sites.^18^ The dimeric nature of RirA may be the source of the observed cooperativity.^43^ For example, binding of the recognition helix of one RirA protomer might cause a conformational change in the DNA or protein that increases the affinity of the recognition helix of the second protomer. We note that N8D RirA exhibited stronger cooperativity than wild-type RirA, and that the cluster coordination in this variant is predicted to be across the two RirA promoters (as in NsrR^15^), perhaps enhancing communication across them. Further studies are needed to explore the nature of the binding and cooperativity.

*In vivo* experiments indicated that N8D RirA was able to bind to IRO sequences and repress the transcription of *vbsC*, involved in synthesis of the siderophore vicibactin. CAS plate assays confirmed that vicibactin was not produced when N8D RirA was introduced to a *rirA*^−^ mutant. However, N8D RirA was completely insensitive to iron levels, as transcription of *vbsC* remained off under low iron conditions.

The N8C variant was found to bind a [4Fe–4S] cluster with enhanced stability compared to wild type, but with lower stability than N8D RirA. This is consistent with the modelling of the N8C RirA structure, based on that of NsrR,^15^ which suggested that the Cys8 residue would not be able to coordinate the [4Fe–4S] cluster without significant conformational change. One possibility would be movement of the cluster (together with the loop carrying the three Cys ligands in the wild-type protein). An alternative possibility is movement of α-helix 1, the N-terminus of which was found to interact with the phosphate backbone of the DNA in the NsrR/*hmpA1* complex.^42^ The shift of helix 1 should also trigger a movement of the two following helices of the DNA-binding helix-turn-helix motif, because it extensively interacts with them in the closely related NsrR structure.^15,42^ A rigid body movement of the three helices together would explain the lowered DNA-binding affinity of the N8C variant compared to wild-type and N8D RirA proteins.

In contrast to N8D RirA, the N8C variant still underwent the conversion from [4Fe–4S] to [2Fe–2S], as observed in wild-type RirA. However, compared to wild-type RirA, conversion occurred at a much slower rate under anaerobic conditions. Conversely, under aerobic conditions, conversion occurred rapidly in comparison to wild-type RirA, but the [2Fe–2S] form was then relatively stable. The facile conversion to a [2Fe–2S] form under aerobic conditions might reflect a conformation that is better suited to coordination of this cluster type, which requires a rearrangement of two of the Cys thiols.^44^ We note that O_2_-mediated rapid conversion of [4Fe–4S] NsrR to [2Fe–2S] NsrR was previously reported for a low molecular weight thiol-coordinated form of [4Fe–4S] NsrR upon exposure to O_2_.^4^

N8C [4Fe–4S] RirA retained the ability to bind DNA, albeit with lower affinity than the [4Fe–4S] forms of wild-type or N8D RirA. The N8C [2Fe–2S] form, which is generated rapidly in the presence of O_2_, was also able to bind DNA, with somewhat higher affinity than the [4Fe–4S] form, but still lower than that of wild-type [4Fe–4S] RirA (Fig. S8†). Thus, a lower degree of repression would be expected *in vivo* for aerobically grown cultures, as observed. However, the DNA-binding affinity of apo N8C was similar to that of the [2Fe–2S] form, suggesting, based on affinities, that de-repression under iron limitation would not be expected. De-repression was observed, however, pointing to additional factors that may affect regulation *in vivo*.

One possible additional regulatory mechanism that could be important when comparing *in vivo* and *in vitro* data is that, as has been observed for FNR,^45^ the apo form may be more susceptible to protease-mediated degradation, which would contribute to de-repression of expression. Degradation of the α-proteobacteria global iron regulator Irr in response to iron levels, mediated by protein oxidation, has also been reported,^46^ and proteolysis also plays a regulatory role in other complex pathways, including Isc Fe–S cluster biogenesis.^47^ Degradation of RirA could also explain why a relatively modest 3- to 5-fold difference in affinity between cluster-bound and apo RirA is sufficient for *in vivo* regulation. While DNA affinities may have evolved to be delicately poised to enable regulation in the correct physiological iron concentration range, some degree of degradation of apo RirA might also be important. We note that a truncated form of RirA was observed upon over-production of RirA in *E. coli*,^8^ and proteolytic degradation could account for why de-repression of N8C RirA-mediated regulation was observed upon iron limitation when affinities alone suggested it would not be. However, we also note that apo RirA continued to partially repress RirA-regulated genes (Fig. 9B), because the complete absence of RirA in the *rirA* mutant resulted in significantly greater depression than observed for wild-type protein under iron limitation.

**Fig. 10:**
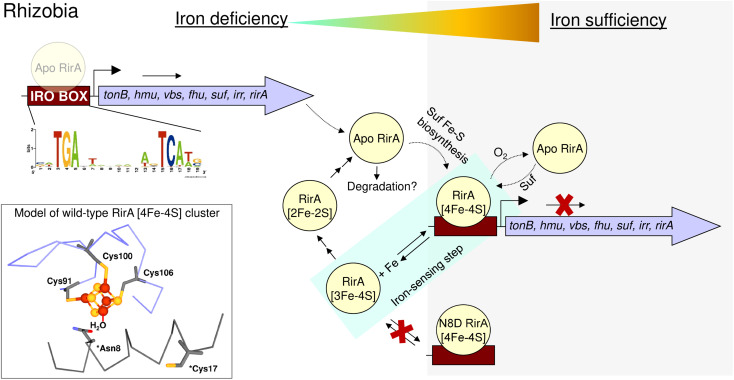
Schematic summary of the proposed mechanism of RirA-mediated regulation of Fe-responsive genes in *Rhizobia*. Under iron sufficiency (grey background), RirA binds a [4Fe–4S] cluster (made *via* the Suf system) and associates with the IRO motif (sequence shown) of the promoter regions of RirA-regulated genes, resulting in repression of transcription. RirA regulated genes include those indicated: *tonB* (energy transducer for iron uptake), *hmu* (heme uptake) *vbs* and *fhu* (for the synthesis and uptake of the vicibactin siderophore, respectively), *suf* (iron-sulfur cluster biosynthesis), *irr* (a second global iron regulator) and *rirA*. Under iron deficiency (white background), the [4Fe–4S] cluster of RirA initially loses a Fe^2+^ ion to form an unstable [3Fe–4S] cluster that degrades to a [2Fe–2S] form and eventually to apo RirA. Forms of RirA containing a degraded Fe–S cluster, or lacking a cluster entirely, bind the IRO motif with lower affinity, and may also be subject to degradation, resulting in increased expression. O_2_/oxidative stress destabilises the RirA cluster, leading to increased turnover of [4Fe–4S] RirA even under iron sufficiency. Figure adapted from ref. 8.

In summary, the work described here supports the proposal that the RirA [4Fe–4S] cluster does not have a fourth amino acid side chain ligand, and highlights the importance of the labile iron for its *in vivo* function. Loss of that iron, which occurs when the metal is scarce, initiates the cluster degradation process, leading to a loss of DNA binding and de-repression of genes necessary for iron acquisition by the *Rhizobium* cell (Fig. 10). The introduction of a potential fourth side chain cluster ligand at position 8 (Asp in place of Asn), did not affect the conformation of the protein, as indicated by the lack of an effect on DNA binding. However, it did significantly reduce the lability of the fourth iron and, with it, the ability of the cluster to degrade under low-iron conditions. Thus, this single substitution abolished the ability of RirA to sense iron, and, consequently, *vbsC* transcription was repressed irrespective of iron availability. The increased stability of the N8D RirA protein may facilitate its structural characterisation, which has not yet been possible for the fragile wild-type [4Fe–4S] RirA protein. Attempts to crystallise N8D RirA in free and DNA-bound forms are ongoing. We note that many of the residues identified in the DNA-bound structure of NsrR^42^ as directly contacting the *hmpA1* DNA are not conserved in RirA, suggesting significant differences in the DNA-binding modes of the two sensors. Finally, the work described here illustrates the high sensitivity of Rrf2 superfamily structure–function relationships; a single substitution converted [4Fe–4S] RirA into a more stable protein that can no longer sense iron, and which has greater similarities to [4Fe–4S] NsrR.^4^

## Data availability

Data supporting the conclusions of this study are available in the main paper, with sequence analyses and additional experimental data included in the ESI.†

## Author contributions

EG, MYYS, JCC, JCF-C, JDT and NLB conceived the study and contributed to experimental design. EG and MYYS contributed to protein production and purification and carried out the spectroscopic and MS experiments with advice from JCC and analysed the data. EG, RD, CS and MIH carried out the SPR experiments and analysed the data. AV and JCF-C carried out the *in silico* modelling. LH and JDT carried out the *in vivo* work and analysed the data. EG, MYYS and NLB wrote the paper with assistance from all authors and particularly JCC, AWBJ, JDT, AV and JCF-C. All authors have approved the final version of the manuscript.

## Conflicts of interest

The authors have no conflicts to declare.

## Supplementary Material

SC-014-D3SC03020B-s001
