# Supplementary material for: Stabilisation of the RirA [4Fe–4S] cluster results in loss of iron-sensing function
Source: Chem Sci. 2023 Aug 22;14(36):9744–58. doi: 10.1039/d3sc03020b (PMC10510648; doi:10.1039/d3sc03020b)
Supplement: SC-014-D3SC03020B-s001 [file SC-014-D3SC03020B-s001.pdf]

Electronic Supplementary Material (ESI) for Chemical Science.
This journal is © The Royal Society of Chemistry 2023

**Supporting Information**

# Stabilisation of the RirA [4Fe-4S] cluster results in loss of iron-sensing function

Elizabeth Gray, Melissa Y. Y. Stewart, Libby Hanwell, Jason C. Crack, Rebecca Devine, Clare Stevenson, Anne Volbeda, Andrew W. B. Johnston, Juan C. Fontecilla-Camps, Matthew I. Hutchings, Jonathan D. Todd, and Nick E. Le Brun

## Supporting Figures

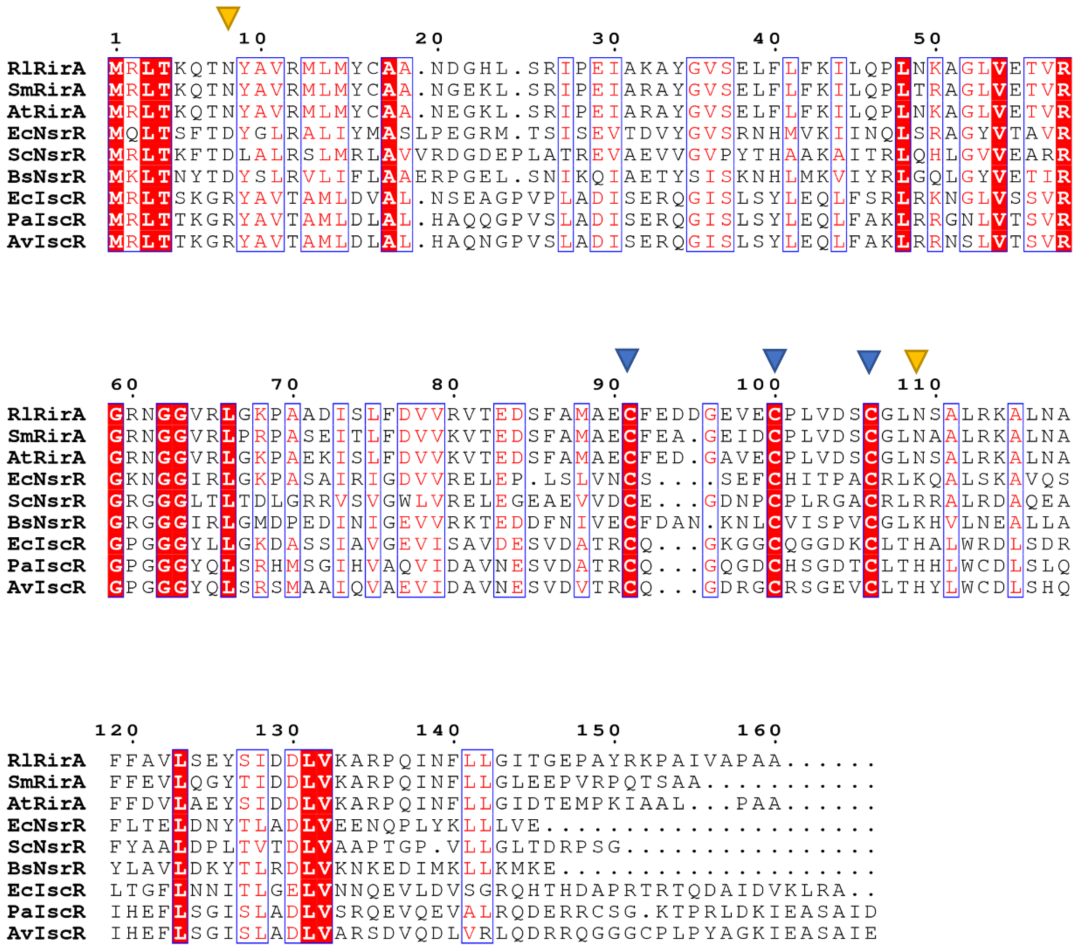


**Figure S1. Alignment of RirA proteins with other NsrR and IscR Rrf2 family regulators.** Alignment of *R. leguminosarum* RirA (RlRirA, Accession number CAC35510) with RirA sequences from *Sinorhizobium/Ensifer meliloti* (SmRirA, WP_003527122) and *Agrobacterium tumefaciens* (AtRirA, WP_003514531), NsrR sequences from *Streptomyces coelicolor* (ScNsrR, WP_011031657), *Escherichia coli* (EcNsrR, WP_032251176) and *Bacillus subtilis* (BsNsrR, WP_063334953), and IscR sequences from *E. coli* (EcIscR, WP_053285796), *Pseudomonas aeruginosa* (PaIscR, WP_034033784) and *Azotobacter vinelandii* (AvIscR, WP_012702552). The three conserved cysteine residues shown/predicted to ligate iron-sulfur clusters in Rrf2 family regulators are indicated by blue arrowheads; other coordinating residues are indicated by yellow arrowheads (Asp8 in NsrR, and His107 in IscR). Blue boxes indicate more than 70% of residues are similar in terms of their physico-chemical properties. The alignment was carried out using Clustal Omega[1] and rendered using ESPript 3.0[2]. Figure adapted from ref[3].

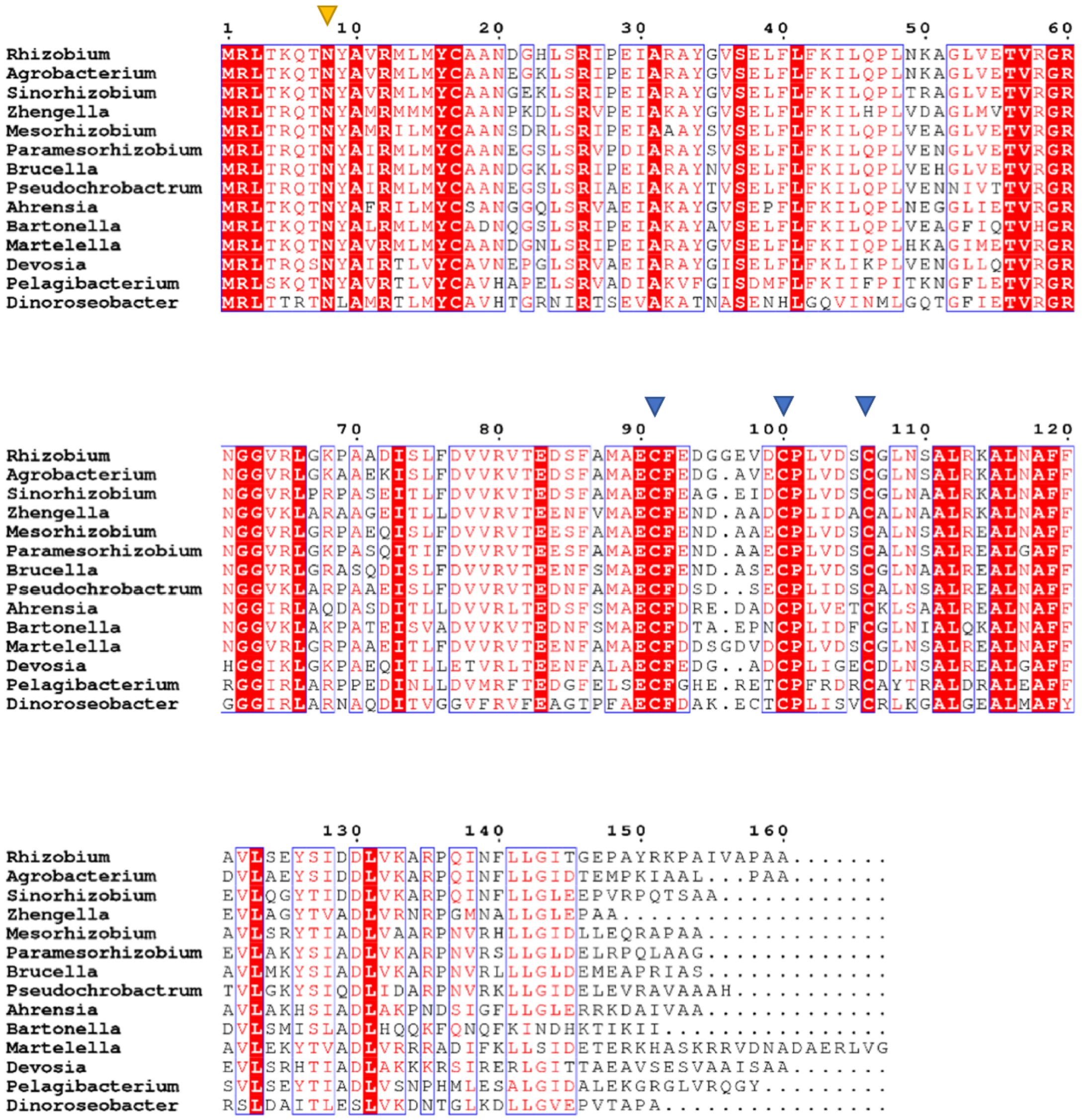


**Figure S2. Alignment of RirA homologues.** Alignment of selected RirA homologues from all RirA-containing families of the order Rhizobiales (Hyphomicrobiales). RirA proteins from: *Rhizobium. leguminosarum* (Accession number WP_222057241); *Agrobacterium tumefaciens* (WP_154961378); *Sinorhizobium meliloti* (WP_003527122), *Zhengella mangrove* (WP_099307082); *Mesorhizobium tamadayense* (WP_125001828); *Paramesorhizobium deserti* (WP_068881613); *Brucella melitensis* (QRN89794), *Pseudochrobactrum saccharolyticum* (KAB0539083); *Ahrensia* sp. (MAZ15170); *Bartonella vinsonii* (WP_208438375); *Martelella alba* (WP_141150609); *Devosia equisanguinis* (WP_126149862); *Pelagibacterium halotolerans* (WP_014129322); *Dinoroseobacter shibae* (ABV93402). The three conserved cysteine residues shown/predicted to ligate iron-sulfur clusters in RirA are indicated by blue arrowheads; position 8 that features a coordinating Asp residue in the related NsrR regulator is indicated by a yellow arrowhead. Blue boxes indicate more than 70% of residues are similar in terms of their physico-chemical properties. The alignment was carried out using Clustal Omega[1] and rendered using ESPript 3.0[2].

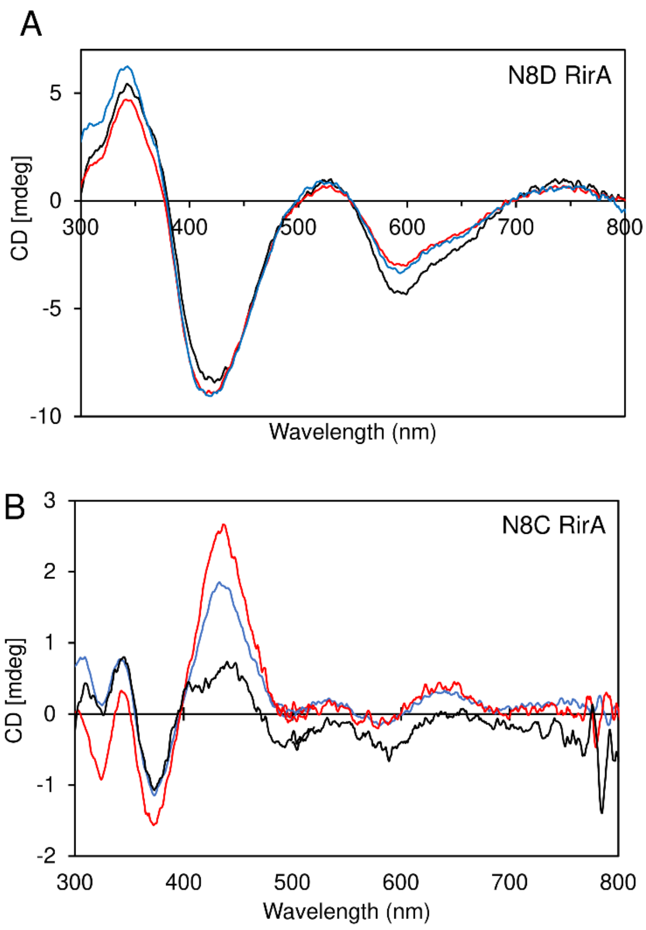


**Figure S3. CD spectroscopy characterisation of N8 variants of RirA following purification and *in vitro* cluster reconstitution.** (**A**) Normalised CD spectra of as-isolated N8D RirA (black line), reconstituted N8C RirA before (blue line) and after (red line) gel filtration. (**B**) As in (A) but for N8C RirA. Spectra before and after reconstitution and gel filtration were very similar, consistent with the stability of the cluster in these variant RirA proteins. Spectra were recorded for as-isolated and reconstituted N8 variant RirA proteins in 25 mM Hepes, 2.5 mM $CaCl_2$, 50 mM NaCl, 750 mM KCl, pH 7.5. .Gel filtered proteins were in 25 mM Hepes, 2.5 mM $CaCl_2$, 50 mM NaCl, 333 mM KCl, pH 7.5. Measurements were obtained using a 1 cm pathlength sealed anaerobic cuvette.

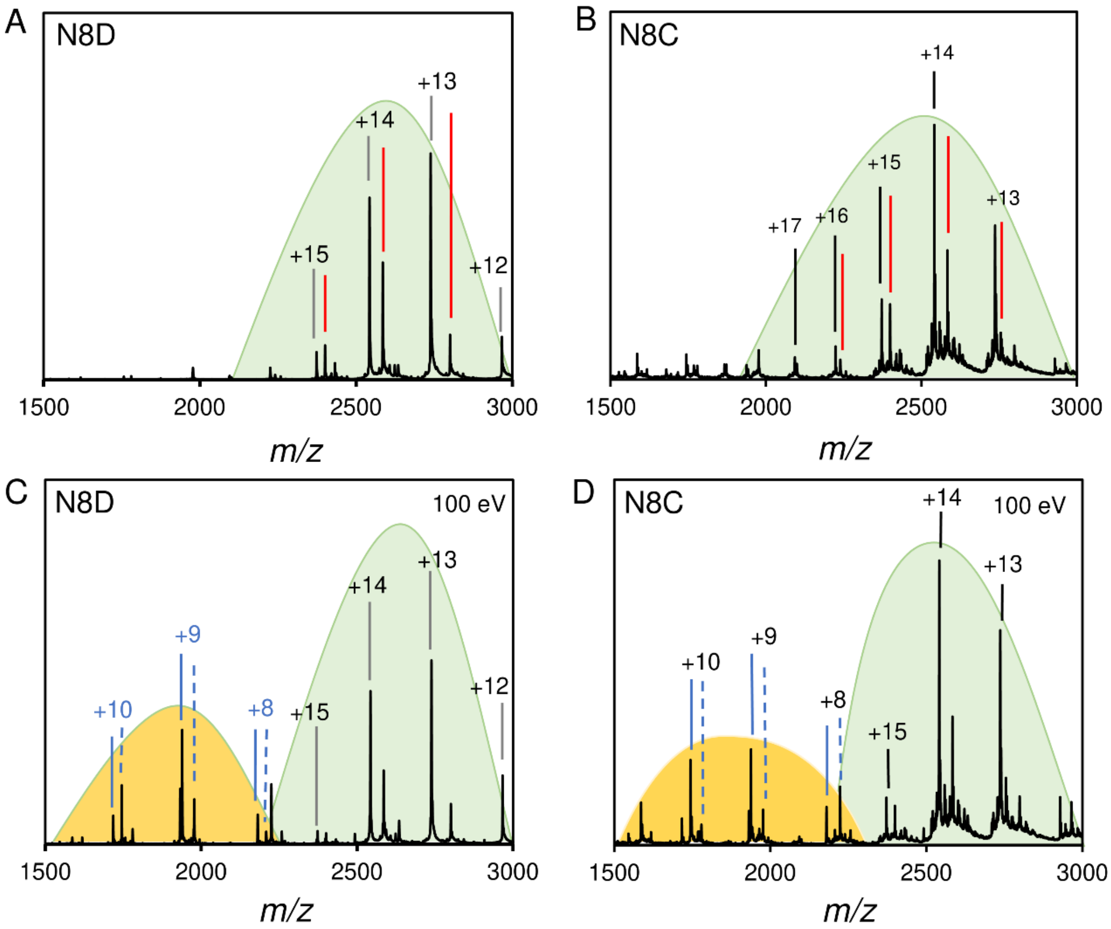


**Figure S4. Non-denaturing ESI-MS characterisation of reconstituted N8D and N8C RirA proteins.** (**A**) *m/z* spectrum of reconstituted N8D RirA under non-denaturing conditions showing peaks due to charge states in the dimer region (green shading). Peaks correspond to two major species: full length N8D RirA (grey lines); and, a truncated version of N8D RirA (red lines) [3]. (**B**) *m/z* spectrum of reconstituted N8C RirA under non-denaturing conditions showing peaks due to charge states in the dimer region (green shading). Peaks correspond to two major species: full length N8C RirA (grey lines); and, a truncated version of N8C RirA (red lines) [3]. (**C**) *m/z* spectrum of N8D RirA upon application of 100 eV in source CID, resulting in a bimodal charge state distribution with charge states corresponding to monomeric (yellow shading) and dimeric (green shading) N8D RirA. Monomeric charge states of +10, +9 and +8 are shown in blue, with peaks corresponding to apo- (solid line) and holo- (dashed line) charge states. Dimeric charge states +15, +14, +13 and +12 are indicated by black lines. (**D**) *m/z* spectrum of N8C RirA upon application of 100 eV in source CID, resulting in a bimodal charge state distribution with charge states corresponding to monomeric (yellow shading) and dimeric (green shading) N8C RirA. Monomeric charge states of +10, +9 and +8 are shown in blue, with peaks corresponding to apo- (solid line) and holo- (dashed line) charge states. Dimeric charge states +15, +14, and +13 are indicated by black lines. N8D and N8C RirA proteins (28 µM in cluster, fully cluster-loaded) were in 250 mM ammonium acetate, pH 7.3.

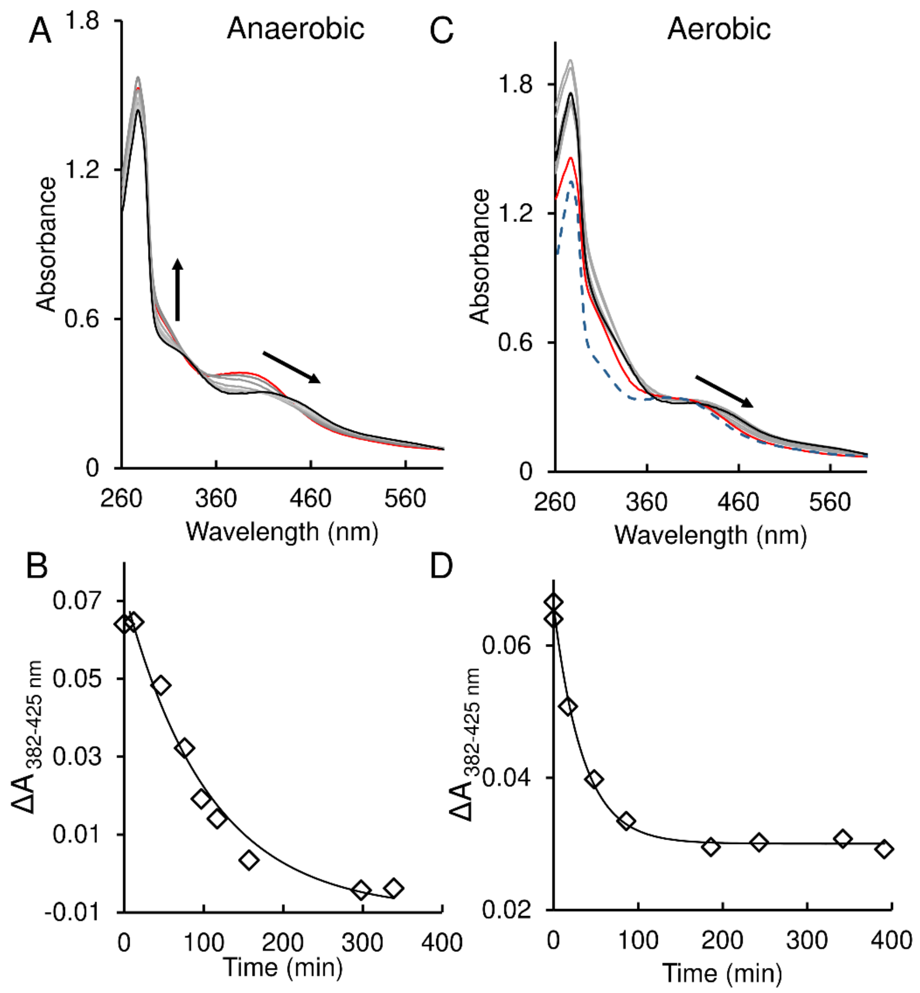


**Figure S5. Time dependence of wild type [4Fe-4S] to [2Fe-2S] conversion promoted by 1 mM EDTA.** (**A**) UV-visible absorbance spectra of wild-type RirA over several hours following the addition of 1 mM EDTA, under anaerobic conditions. Initial spectrum is shown in red, all intervening spectra are in grey, and end point spectrum (after 350 mins) is in black. Arrows indicate direction of absorbance change. RirA (29 μM) was in 25 mM Hepes, 2.5 mM $CaCl_2$ 50 mM NaCl, 750 mM KCl, pH 7.5. (**B**) Plot of absorbance obtained for anaerobic data: $A_{382\ nm} – A_{425\ nm}$ as a function of time, solid line represents a fit of a single exponential function to the data ($k$ = 0.009 min $^{-1}$). (**C**) UV-visible absorbance spectra recorded of wild-type RirA following addition of 1 mM EDTA in aerobic conditions (in presence of 230 μM $O_2$). The initial spectrum prior to $O_2$ exposure is a blue dashed line, the initial spectrum following exposure to $O_2$ is in red, all intervening spectra are in grey, and the end point spectrum is in black. Arrows indicate direction of absorbance change. RirA (28 μM in cluster following reconstitution) was in 25 mM Hepes, 2.5 mM $CaCl_2$ 50 mM NaCl, 750 mM KCl, pH 7.5. (**D**) Plot of absorbance obtained from aerobic data: $A_{382\ nm} – A_{425\ nm}$ as a function of time. The solid line represents a fit to the data following exposure to $O_2$ ($k$ = 0.029 min $^{-1}$), as in (B). The measurements were obtained using a 1 cm pathlength sealed anaerobic quartz cuvette.

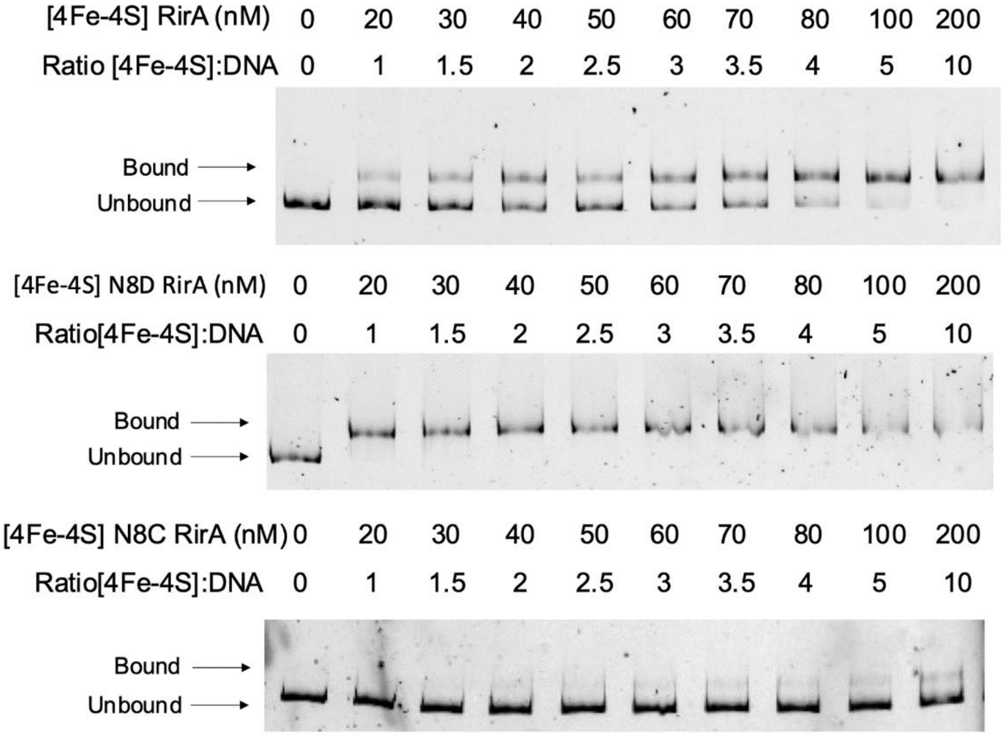


**Figure S6. Binding of wild type, N8D and N8C [4Fe-4S] RirA proteins to the *fhuA* promoter probed by EMSA.** Electrophoretic mobility shift assays of: (**A**) wild-type [4Fe-4S] RirA; (**B**) N8D [4Fe-4S] RirA; (**C**) [4Fe-4S] N8C; to 20 nM of the *R. leguminosarum fhuA* promoter containing the IRO box sequence `GGTGACTAAAATAATCATC` [3]. The ratios of protein to DNA, and the bound and unbound states of DNA are indicated. The binding buffer contained 10 mM Hepes, 50 mM NaCl, 2.5 % sucrose, 2.5 mM $MgCl_2$, 1 mM DTT, pH 7.9. We note that although EMSAs are often used to qualitatively and quantitatively assess protein-DNA binding, there are some inherent disadvantages that makes, particularly quantitative, characterisation unreliable. For example, during the electrophoresis experiment where the protein-DNA complex runs through the gel at a different rate to the uncomplexed DNA/protein, bound and unbound species are not in true equilibrium, leading to over-estimation of the dissociation constant ($K_d$). Alternatively, some complexes may be more stable under conditions of the gel-based experiment than they are in a free solution[4]. For protein-DNA interactions that are dependent on an environment-sensitive Fe-S cluster, EMSAs are particularly problematic. The EMSA running buffer contains metal chelator and the electrophoresis experiment produces $O_2$, both of which potentially destabilise the Fe-S cluster, again leading to possible over-estimation of the $K_d$. Thus, although EMSAs generally give dependable qualitative indication of (relative) binding, absolute binding affinities are better determined by more sensitive analytical methods such as spectroscopy or surface plasmon resonance (SPR), which are equilibrium methods. SPR, in particular, can be performed rapidly, and so issues with degradation are expected to be less troublesome. Thus binding affinities determined by SPR are generally more reliable than those determined by EMSA.

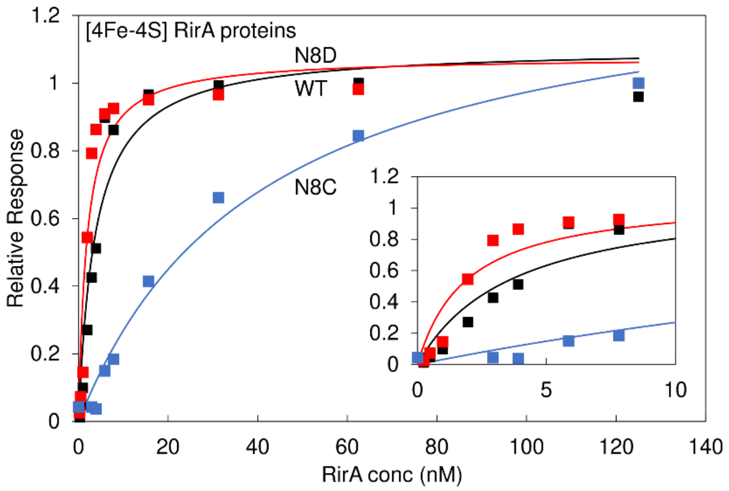


**Figure S7. Binding of wild-type, N8D and N8C [4Fe-4S] RirA proteins to the *fhuA* promoter probed by SPR.** Data as in Figure 8 of the main paper but fitted to a simple binding equation. Inset shows the early part of the titrations in more detail. Quality of the fits was deemed unsatisfactory for wild-type and N8D RirA datasets, particularly in the early part of the titration where the sigmoidal nature is not simulated. $R^2$ values, as a measure of goodness of fit, were 0.940 and 0.931 for wild-type and N8C proteins, respectively. Fits to the Hill equation (Fig. 8) gave satisfactory fits with $R^2$ values of 0.988 and 0.997, respectively. N8C RirA data were fitted reasonably well by the simple binding equation, $R^2$ = 0.981, but the goodness of fit was also improved using the Hill equation ($R^2$ = 0.994). This reflected a lower level of cooperativity for N8C compared to N8D or wild-type RirA.

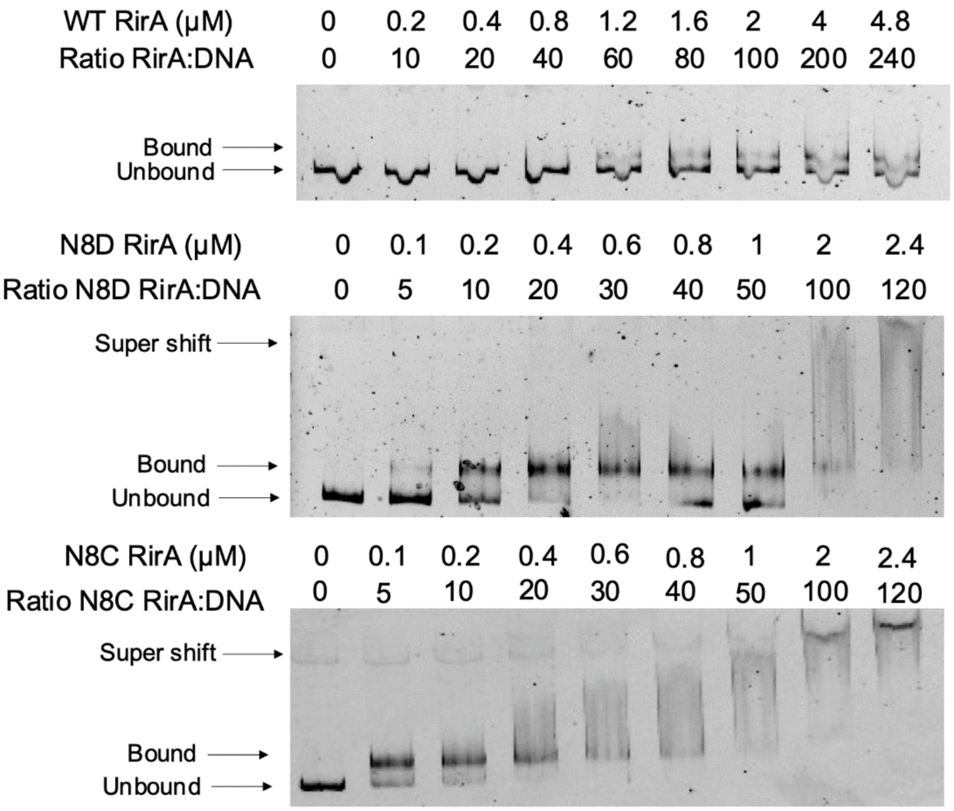


**Figure S8. Binding of wild type, N8D and N8C apo RirA proteins to the *fhuA* promoter probed by EMSA.** Electrophoretic mobility shift assays of: (**A**) apo wild type RirA; (**B**) apo N8D RirA; (**C**) apo-N8C RirA; to 20 nM of the *R. leguminosarum fhuA* promoter containing the RirA consensus sequence. Slow running and sometimes smeared bands are labelled with 'super shift', indicating that they arise from non-specific binding of protein at high protein:DNA ratios. The ratios of protein to DNA, and the bound and unbound states of DNA are indicated. The binding buffer contained 10 mM Hepes, 50 mM NaCl, 2.5 % sucrose, 2.5 mM $MgCl_2$, 1 mM DTT, pH 7.9.

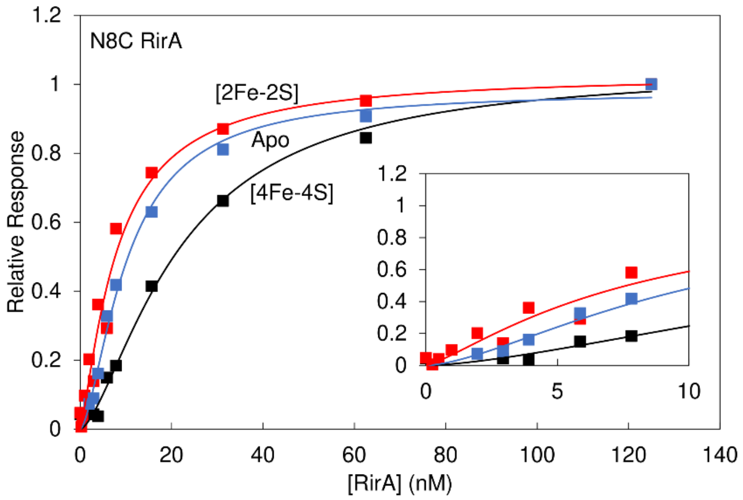


**Figure S9. Binding of [2Fe-2S], [4Fe-4S] and apo N8C RirA to the *fhuA* promoter probed by SPR.** Analyte binding response of [2Fe-2S] (blue), [4Fe-4S] (black) and apo- (red) N8C RirA to 200 nM 30 bp *fhuA* promoter region containing the RirA consensus sequence. For the cluster-bound forms, 0 - 125 nM dimeric protein concentration was incubated with 200 nM of the 30 bp *fhuA* oligo in 10 mM Hepes, 150 mM NaCl, 0.05% Tween, pH 7.4. Apo-NC8 RirA was prepared in 5 mM EDTA overnight, and then binding reactions were performed as for holo-proteins.

**Supporting References**


1. F. Sievers, A. Wilm, D. Dineen, T. J. Gibson, K. Karplus, W. Li, R. Lopez, H. McWilliam, M. Remmert, J. Soding, J. D. Thompson and D. G. Higgins, *Mol. Sys. Biol.*, 2011, **7**, 539.
2. X. Robert and P. Gouet, *Nucl. Ac. Res.*, 2014, **42**, W320-324.
3. M. T. Pellicer Martinez, A. B. Martinez, J. C. Crack, J. D. Holmes, D. A. Svistunenko, A. W. B. Johnston, M. R. Cheesman, J. D. Todd and N. E. Le Brun, *Chem. Sci.*, 2017, **8**, 8451-8463.
4. R. A. C. Ferraz, A. L. G. Lopes, J. A. F. da Silva, D. F. V. Moreira, M. J. N. Ferreira and S. V. de Almeida Coimbra, *Plant Meth.*, 2021, **17**, 82.
